# Analyzing Lipid Membrane Defects via a Coarse-Grained to Triangulated Surface Map: The Role of Lipid Order and Local Curvature in Molecular Binding

**DOI:** 10.1021/acs.jctc.4c00082

**Published:** 2024-03-27

**Authors:** Rianne W. I. van der Pol, Bregje W. Brinkmann, G. J. Agur Sevink

**Affiliations:** † Leiden Institute of Chemistry, Leiden University, P.O. Box 9502, 2300 RA Leiden, The Netherlands; ‡ Institute of Environmental Sciences, Leiden University, P.O. Box 9502, 2300 RA Leiden, The Netherlands

## Abstract

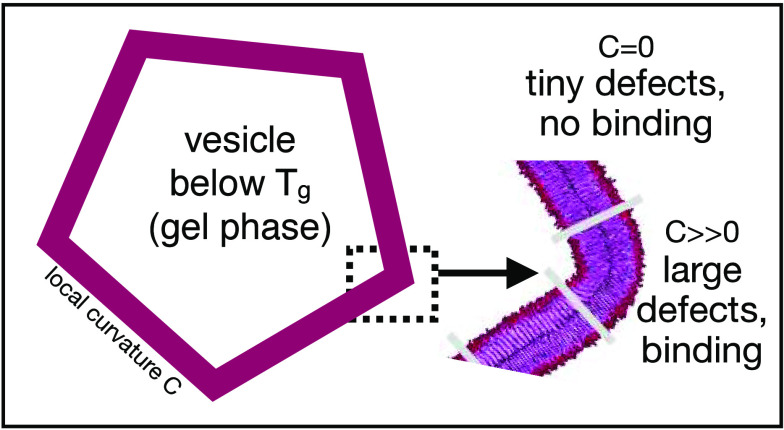

Lipid packing defects are known to serve as quantitative indicators for protein binding to lipid membranes. This paper presents a protocol for mapping molecular lipid detail onto a triangulated continuum leaflet representation. Besides establishing the desired forward counterpart to the existing inverse TS2CG map, this coarse-grained to triangulated surface (CG2TS) map enables straightforward extraction of the defect characteristics for any membrane geometry found in nature. We have applied our protocol to investigate the role of local curvature and varying lipid packing on the defect constant π. We find that the defect size is greatly influenced by both factors, arguing strongly against the usual assignment of a single defect constant in the case of more realistic membrane conditions. An important discovery is that lipids in the gel phase produce larger defects, or a higher π, in domains of high (local) curvature than the same lipid in a liquid phase of any curvature. This finding suggests that membranes featuring very ordered lipid packing can bind proteins via large defects in curved regions. Finally, we propose a route for estimating defect constants directly from the standard membrane properties. Identifying the precise role of composition, lipid (tail) order, and (local) curvature in defects for the irregular lipid structures that are (temporally) present in many biological processes is instrumental for obtaining fundamental insight as well as for a rational design of membrane binding targets.

## Introduction

Defects play an important role in many functional materials, and obtaining control over their density, structure, and connectivity is a long-standing challenge for the rational design of nanostructured materials. For instance, the ability to generate block copolymer materials containing only few defects, or only defects of a particular nature, is crucial for applications in nanolithography. One of the key concerns in gaining control is the considerable stability of oppositely charged (topological) defects, particularly in the absence of externally applied modulating fields. Such defects, which can be seen as quasi-particles, only weakly interact over large distances, rendering defect annihilation generally a very slow process. Also the biophysical modeling community is increasingly studying the role of defects in regulating structure and function in biological systems. As an example, a recent study identified a distinct mechanical coupling between defects and curvature that signifies the role of topological defects in the creation of sharp features in developing embryos.^1^ While some defects can thus be classified as the classical (topological) type, the physical origin and functional role can also be quite different. Adding such context, for instance, by rebranding membrane defects as surface accessible hydrophobic area (SAHA), has already been proposed. For consistency, however, we used the standard notation in this study.

Focusing on (structural) defects in biomembranes, where features stem from membrane composition and interaction/embedding in a surrounding biomatrix, it has become apparent that the experimentally observed differential recruitment of membrane-binding peptides and proteins is strongly regulated by lipid packing defects, since they introduce or enhance exposure of the hydrophobic membrane core to hydrophobic binding domains. Particularly, the increase in packing defects with membrane curvature can be interpreted to reduce the (energetic) barrier for partial insertion of a particular class of proteins and thus introduces a curvature preference that is known as *sensing*. Consequently, the natural separation between proteins that bind to a membrane (“binders”) and proteins that bind to a strongly curved membrane (“sensors”) can be computationally analyzed via defect characteristics.^2^ Such defects, which originate from the conformational dynamics of lipids that together constitute the membrane, appear to be of a very local nature. In particular, the defect size distribution on a 2D lattice was previously found to agree well with that of a percolation model, i.e. displaying single-exponential decay with size, implying that lipid defects are transient and, in principle, noninteracting, with larger defects being generated by coalescence of noninteracting smaller defects.^3^ Larger defects on curved membranes were identified to promote folding of the considered binding domain within proteins, with the defect structure clearly responding to the bound protein, while smaller defects on flat membranes were found to inhibit folding of the same protein domain.^3^

The functional role of defects in important mechanisms such as molecular recognition renders the quantification of key defect properties for membranes of arbitrary shape of some importance, especially those with varying local compositions and curvatures along the lipid–water interface that are present in cellular structures. In practice, however, defects fall outside the class of basic membrane attributes or membrane *descriptors* that are routinely analyzed, which usually concern properties that are averaged over the entire (closed) membrane, including the area per lipid (*A*_*l*_), membrane thickness (*d*_*HH*_), (orientational) order parameters, and several others.^4−8^ Moreover, as most of the standard tools for determining these basic properties cannot deal with curvature, it is advisible to employ recent special tools such as the Fast Analysis Toolbox for Simulations of Lipid Membranes (FatSLim)^9^ and Surface Assessment Via grid Evaluation (SuAVE)^10^ if one is interested in computing sensible values for curved membranes.^11^ PackMem, the only publicly available tool for extracting lipid packing defects characteristics, restricts itself to planar geometries, see the PackMem literature for details, albeit that also geometries featuring constant curvature like tubes or spheres have been considered by the developers using special private routines.^12,13^ In line with the percolation model, Packmem analyzes three types of defects - shallow, deep and overall - in terms of their scaling coefficient π (in units of Å^2^), conveniently called the defect size constant, by fitting the normalized frequency of finding a defect of that type and size A in a simulation trajectory to the monoexponential decay function
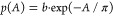
1where the area *A* is in units of Å^2^. As such, the magnitude of π can thus be interpreted as the average size of the lipid-packing defects in the entire membrane. For completeness, we mention a recent development that approaches the challenge of analyzing irregular membranes from a different angle, by identifying lipid packing defects as voids in three dimensions via a grid-based free volume method.^14^ Besides the observation that the defect characterization of this 3D method differs from the projection approach used by Packmem and this study, the method produces trends that are in full agreement with Packmem. For this reason, we compare our results only to the latter method.

The goal of the current study is twofold. First, we develop a Coarse-Grained to Triangular Surface (CG2TS) mapping algorithm that transforms positions of specified atom or particle types from simulation results directly into the best matching triangulated surface. As such, this algorithm is applicable to membranes of arbitrary resolution, shape, topology, and composition. Next, we refine this 2D triangulated lattice until the tiling is dense enough for extracting defects and defect (size) constants. Starting from this stage, we adopted the general rules introduced by PackMem. Our procedure has the advantage that no predefined symmetry is needed and thus that it can deal with arbitrary local curvatures and (convex) membrane shapes, where the role of defects is expected to be most significant. In addition, by coupling this procedure to lattice attributes like local curvature, we may directly analyze the relation between local curvature and the associated defect constant along the membrane. Second, and an incentive for future research, the triangulated surfaces obtained by CG2TS can be directly exploited as a starting point for continuum treatments. For more details on this feature, we refer to the Discussion section.

This study is structured as follows: first, we will shortly discuss the CG2TS concept, referring to the Methods section for algorithmic detail. To validate the equivalence between using a triangular and a square lattice, which are known to provide different critical exponents in percolation theory,^15^ we compare results of the CG2TS-based procedure to PackMem results for two one-component membranes and two well-chosen mixed membranes in a flat configuration. Having validated our new procedure, we evaluated how membrane composition and phase behavior affect defect characteristics. Consequently, we turn to buckled membranes for an investigation of how emerging local properties such as tension and curvature regulate defects, and we identify a significantly enhanced effect of local curvature on defects in the gel phase when compared to the liquid phase. This is an intriguing finding since order and disorder are indeed thought to be among the key factors in regulating the biological function of lipids. Finally, we apply our method to analyze the defect characteristics for two exotic membrane shapes of biological relevance.

## Methods

The code for CG2TS is written in Python 3.7 and builds upon the open source libraries MDAnalysis,^16,17^ Numpy, SKLearn, Open3D, and Optimesh. In this section, we discuss the CG2TS implementation and the defect analysis separately as they are set up as two different classes. Additional technical details are provided in the Supporting Information (SI). As a general note, it is important to realize that the criteria employed for defect assignment after the projection of particle coordinates to a mesh are the same in our protocol and in PackMem. The important difference is in the projection since it extends the applicability of the protocol as well as the analysis of defect properties to membranes of arbitrary shape and curvature. Our choice for an unstructured 2D triangular mesh in the CG2TS protocol is inspired by finite elements methods.

### General Outline of the CG2TS Implementation

**Figure 1:**
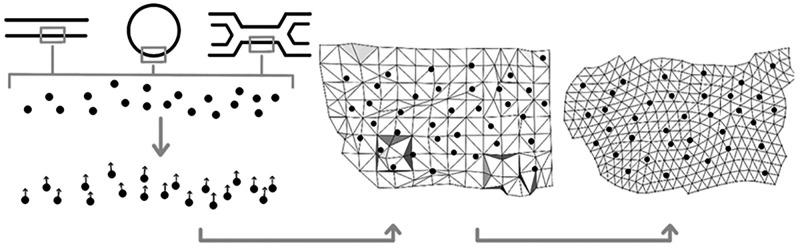
Sketch of the protocol used for obtaining a nearly uniform and dense triangulated surface reconstruction. First, the lipid pool is separated into two leaflets, and one atom or bead of each lipid is selected. For each of these atoms or beads, a normal vector is determined. Following this step, these vectors are used for the construction of a triangulated surface. In the next and final step, the triangular mesh is recasted in the desired form by division and smoothing. The resulting mesh is used for defect selection.

The three consecutive steps required for generating the surface of triangular tiles that serves as a starting point for defect characterization are illustrated in Figure 1. The protocol starts with clustering all lipids in the structure into separate pools for each leaflet. Here, we describe the steps taken for each individual leaflet. First, all lipids are represented by a point position of a bead and an orientation vector. We select the GL2 bead for the position and extract the normal from the local surface spanned by all points in the direct vicinity of the considered point, making sure that all normals correctly point toward lipid tails at the end. The first triangulated surface is constructed by solving a Poisson problem, i.e. determining the scalar indicator function - one inside and zero outside - whose Laplacian equals the divergence of the orientation vector field at the point positions.^18^ This generates a global solution that is smooth and robust to noisy data, which is a great advantage compared with alternative approaches. The triangular mesh that results from the Poission method is generally not evenly distributed in size, so we have to employ an iterative Centroidal Voronoi Tesselation method to resize and smooth the mesh in two consecutive steps.^19^ The resulting final mesh of proper tile sizes is used for the assignment of defects.

### Defect Assignment and Distribution

After CG2TS projection, defects are analyzed using a procedure that is conceptually equivalent to the one employed by Packmem, see ref.^13^ for algorithmic details and illustrations. First, we project all atom/bead coordinates of each lipid along the local (averaged) surface normal onto the triangulated two-dimensional surface mesh that represents the membrane leaflet to which the lipid belongs. For an entirely flat membrane, this would give rise to the orthogonal projection of coordinates employed in the Packmem. Next, we select all tiles (in Packmem: all grid cells) in the mesh that overlap with the particular projected atom or bead. The reference distance, i.e., the Van der Waals radius of this atom or bead, for the coordinates of tile midpoints is specific for the chemical nature of the atom/bead and force field considered; selected tiles that fall within that distance receive a type-specific value. Just like in Packmem, this value (*d*_*type*_) depends on the depth of the considered atom or bead with respect to the reference glycerol and whether the projected atom or bead is aliphatic or polar in nature, with *d*_*type*_ = 1 relating to no defect, *d*_*type*_ = 0 a (deep) defect and, if required, *d*_*type*_ = 0.001 a shallow defect, in full agreement with the Packmem procedure.^13^ To avoid membrane inclusions like proteins playing the role of (large) defects, tiles to which no values are assigned via projection are disregarded. Next, the connectivity of individual defect tiles (*d*_*type*_ < 1) is considered by clustering tiles of *d*_*type*_ < 1 that share a vertex. Yet, if a thus formed defect cluster contains tiles that belong to the outer boundary of the simulation box, boundary effects become eminent, and we remove this cluster from our analysis. Finally, the total area of each cluster is listed in a text file. A Jupyter Notebook code to generate such a text file from a trajectory can be found at GitHub at https://github.com/Asevink/Codes/blob/main/CG2TS.ipynb. This concludes the protocol for defect assignment. Next, binning individual cluster sizes provides a probability distribution, which can be fitted by a single exponential to extract the defect constant π. Introducing a logarithmic-normal rescaling casts the challenge of fitting the simulated distribution into a standard linear regression problem, and all of the usual tools for analysis apply.

### Domain Identification Based on Curvature

For the membranes of varying curvature, such as the buckled and junction membranes, we link each defect along the membrane to the curvature that it is associated with. Instead of particular values, we distinguish only between the sign of the curvature, noting that in most of the considered membrane geometries curvature does not change along one of the Cartesian directions. First, the surface is binned, and normals are averaged over all bins along this invariant direction. The resulting normals per bin are attributed to negative, positive, or flat curved domains depending on the angle between two neighboring bins, see the SI for implementation detail.

### Fitting Procedure

The exponential decay function from percolation theory is only expected to fit the numerically obtained defect area probability distributions in a restricted data range; see the Discussion section. The main challenge is the selection of bounds for an appropriate fitting window. In our case, we allowed the upper and lower bounds to vary between distributions; optimized values are provided in two tables in the SI. We have particularly considered the goodness of the fit (*R*^2^), selecting the range yielding the highest *R*^2^ as the starting point. Diagnostic plots were then employed to verify whether the outcome was in agreement with assumptions made for a linear fit, focusing on the spectrum of the residuals. These criteria include the following: linearity of the data, normally distributed noise with equal variance (homoscedasticity), and checking for outliers with high leverage. Fulfillment was assessed via diagnostic plots: 1) A residual vs fitted plot and the scale-location plot demonstrate the behavior of the residuals. They should exhibit a (near) linear pattern, which indicates that a linear fit is correct. 2) A normal quantile-quantile plot assesses whether the noise is normally distributed. 3) The Cook’s distance and 4) the residual leverage plot identify outliers that exert significant leverage and consequently impact the final fit. Further details about this standard analysis in linear regression can be found in *An Introduction to Statistical Learning*.^20^ In case the criteria were not satisfied, the range was adapted, and all tests were redone. Based on such a recursive analysis of these plots, the appropriate fitting range was determined. All the diagnostics plots for the constants mentioned in this paper are shown in the SI.

### Coarse-Grained Simulations

Although the procedure for defect characterization considers force field parameters such as molecular topology and bead/particle size as input, it is further unaware of how well the coarse-grained lipid representation captures actual membrane structure and dynamics. We considered appropriate representations for the different lipids. For membrane composed of DPPC, POPC or mixtures thereof, we used the most recent Martini 3 force field,^21^ while membranes containing cholesterol, which is not yet well-parametrized in the latest Martini force field, were simulated using Martini 2 instead.^22^ In both cases, Gromacs 2019.3 was used as a simulation engine. The junction membrane, which contains a mixture of DOPC and diacylglycerol (DAG), was simulated with LAMMPS using the SPICA force field (https://www.lammps.org).^23−26^Table 1 shows the composition and settings for each system.

**Table 1:** Composition and Simulation Temperature for All Systems Considered in This Study

name	# dppc	# dopc	# popc	# dlipc	# chol	# dag	*T*
dppc318	318	-	-	-	-	-	303
dppc2858	2858	-	-	-	-	-	303
dppc5080	5080	-	-	-	-	-	303
dppc gel	3286	-	-	-	-	-	285
buckle liq	3287	-	-	-	-	-	325
buckle gel	3287	-	-	-	-	-	285
raft	364	-	-	546	390	-	303
dppc+chol	1164	-	-	-	836	-	303
dlipc+chol	-	-	-	1668	332	-	303
popc	-	-	2858	-	-	-	303
dppc+popc	610	-	610	-	-	-	303
LN	5343	-	-	-	-	24500	303
junction	-	1074	-	-	-	460	303

All setups contain an ion concentration of 0.1 M consisting of Na and Cl ions. With Martini, we used a 20 fs time step. The velocity rescale method was used for the temperature coupling at 303.15 K, albeit that we also considered 285 and 325 K to evaluate defect constants for two different lipid phases. The pressure (with compressibility of 3.0 × 10^–5^ bar, 12 ns) was semi-isotropically coupled with the Parinello-Rahman method. A 1.1 nm cutoff was considered for both the Coulomb interactions, implemented via a reaction field, and the van der Waals interactions, with a shifted Verlet scheme. The neighbor list updates every 20 timesteps. With the SPICA force field, we used a 10 fs time step and an *NP*_*x*_*P*_*z*_*T* ensemble. The temperature and pressure (1 atm) were coupled with a Nosé–Hoover thermostat and barostat, in the x and z directions. The nonbonded interactions were truncated at 15.0 Å. All flat bilayer configurations were set up using the CHARMM-GUI.^27,28^ The spherical lipid nanoparticle was built with Packmol,^29^ with the settings mentioned above, except the pressure coupling, which was isotropic. The total length of all simulation trajectories, i.e. 1 μs, was selected based on the expected relevant time scales in lipid dynamics, and the last 500 ns was used for analysis.

## Results

We start by analyzing whether the connectivity of the two meshes considered, i.e. the Triangular Mesh (TM) obtained after CG2TM projection or the Cubic Mesh (CM) used by Packmem, alters the defect size distributions and fitted packing defect constants π. In the Discussion section, we show that direct comparison is only an option when considering membranes simulated using the same CG force field (FF), so we performed Packmem analysis of our simulation results instead of taking values from the literature.

Initially, we consider flat membranes composed of DPPC lipid since they exhibit a single phase and because a flat geometry is appropriate for both methods. Moreover, phosphatidylcholines are the most common phospholipids in biological membranes,^30^ and DPPC is an important representative of this class. As elastic membranes do feature (transient) curvature formation in the form of capillary waves with fluctuation spectra that are specific to patch size, we simulate different patch dimensions to investigate whether π is sensitive to this factor. At the same time, this setup also provides general insight in the role of finite sampling.

### Flat Membranes: Tail Flexibility and Mixing

**Figure 2:**
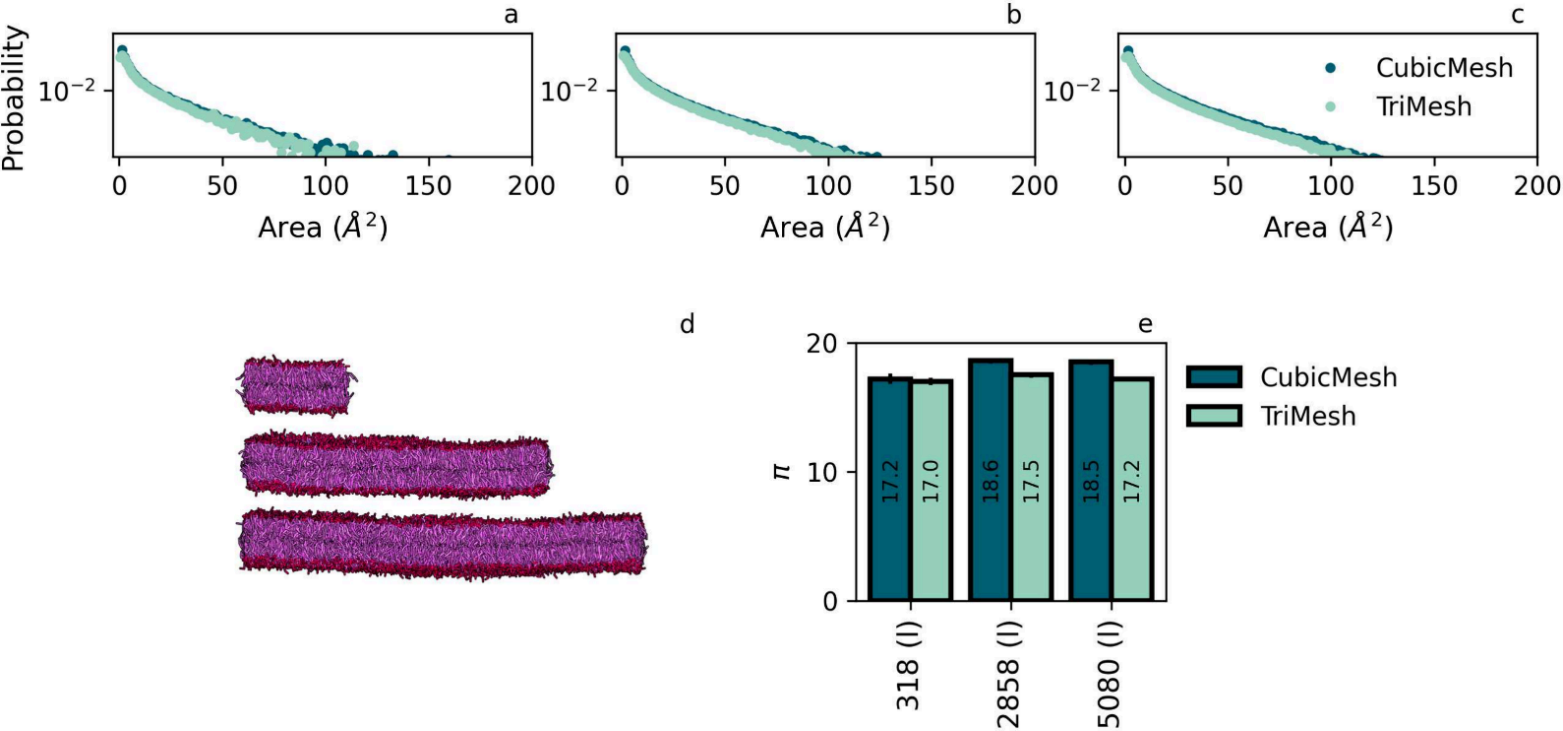
Comparison of defect characteristics obtained using our TriMesh and the CubicMesh of Packmem. We considered one-component membrane patches of DPPC of increasing dimension. Panels a, b, and c show the probability distribution of the defect area with increasing lipid patch size (cyan - our protocol, dark blue - PackMem). Panel d shows a snapshot for the three lipid patches, increasing from 10 × 10 nm, to 30 × 30 nm, and to 40 × 40 nm in size. Panel e shows the π obtained from fitting the distribution shown in parts a-c to a single exponent.

Direct comparison of our results for flat DPPC membranes of increasing patch size, see Figure 2, shows that 1) the distributions of defect areas determined using TM (our protocol) and CM (Packmem) match each other very well for all considered membrane patch dimensions and 2) fitting a single exponential to these distributions shows that patch size does not play a significant role in the value of the defect constants π.

Next, we first concentrate on the comparison of TM and CM. For the smallest DPPC membrane, containing only 159 lipids per leaflet, defect constants obtained by fitting defect size distributions for the two methods show an excellent match: π^CM^ = 17.1 ± 0.3 for a CM and π^TM^ = 17.3 ± 0.4 for our TM protocol. Also increasing the patch size, by adding more DPPC lipids, shows a proper match between the sampling distributions obtained by TM and CM, see Figure 2 for a visual comparison and SI for numerical values. We find that fitted π values for the two different meshes are within a few percent of each other, which we consider a reasonable error margin. In particular, such a margin is also identified for the variation of π with patch size for a fixed CM or TM mesh type.

An important observation is that the quality of the exponential fits improves with increasing patch size, as reflected in higher R^2^ values, see the Discussion section and SI for details about the quality of the fit. This can be understood as follows: ergodicity is required for obtaining a precise exponential decay of the defect distribution that is expected from percolation theory.^3^ Such a situation can be reached by sampling the lipid dynamics via either infinite time traces of smaller membranes or, equivalent, shorter time traces of infinitely large membranes. In practice, however, we determine π from finite sampling data, via a procedure that is prone to fitting issues, and one has to conclude that developing an entirely deterministic procedure is and will remain out of reach. As a result, π-values within a 1–2% range should be regarded equivalent because this is the level of variation identified as typical for different instances of membranes of the same composition. Turning back to our extracted π, the sampling considered in the three patches thus appears to be sufficient for reliably extracting defect constants in all cases. The matching values, between different sizes but also between TM and CM, also provide a hint that (transient) mild curvature due to capillary waves, which perturbs flat membranes in a size-dependent manner, does not play a significant role in the defect characterization. We do observe that π values obtained by the TM protocol appear to be less sensitive to the patch size.

**Figure 3:**
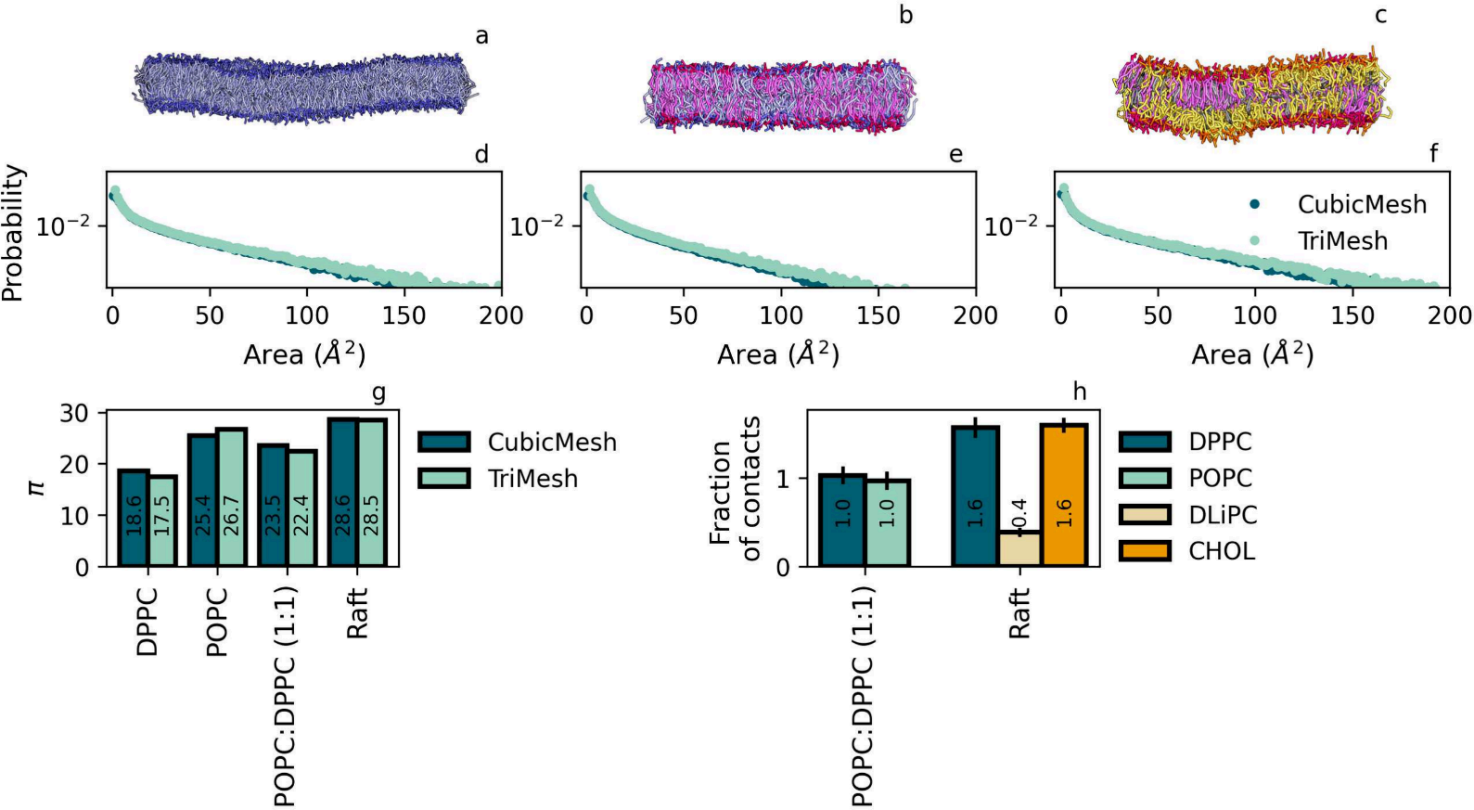
Comparison of TriMesh and CubicMesh for other flat membranes setups. Panel a: snapshot of a membrane composed of monounsaturated POPC, b: snapshot of a mixed membrane (POPC:DPPC (1:1)); and c: snapshot of a raft membrane (DLiPC, DPPC, CHOL). Panels d-f show probability distributions corresponding to the membranes in a-c. Panel g: π obtained from fitting CubicMesh and TriMesh distributions. Panel h: The fraction of contacts with DPPC is within a standard cutoff of 1.1 nm.

Next, we analyze the role of lipid flexibility and domain formation via membrane setups for the largest patch size considered in the previous paragraph. Bond saturation plays a role in conformational sampling, with lipids containing unsaturated bonds like POPC or DLiPC more likely to generate shallow defects as a result of their increased tail flexibility.^13^ Here, we make no distinction between shallow and deep defects, see the discussion later on, but we consider the question whether the overall defect constant allows us to differentiate between DPPC, which bears fully saturated tails, and monounsaturated POPC. In addition, we analyze two multicomponent membranes that mimic important properties in biomembranes: full mixing, using a 1:1 mixture of DPPC and POPC, or raft domain formation by liquid ordered-disordered coexistence (DLiPC:CHOL:DPPC). Mixing or demixing is as usual monitored via the fraction of lipid contacts ϕ,^31^ see the Supporting Information for details, where we have introduced normalization by the concentration of the considered lipid to render ϕ = 1 in the case of ideal mixing and ϕ = 0 for complete demixing. From the calculated values for the mixed system, see Figure 3, we may conclude that DPPC and POPC are indeed fully mixed. For the raft system, we concentrate our analysis on the fraction of contacts with DPPC. It shows that DPPC is significantly more in contact with itself and cholesterol than with DLiPC, containing four unsaturated bonds, stressing a clear separation into relatively pure DLiPC:CHOL and DPPC:CHOL phases.

Figure 3 shows that the defect constant π_POPC_ for the monounsaturated POPC lipid is indeed larger than π_DPPC_ for the fully saturated DPPC, signaling that enhanced tail flexibility indeed gives rise to a higher overall defect constant, i.e. more and larger defects, in agreement with earlier findings by Packmem. Upon mixing POPC with DPPC, the defect constant drops to a value that lies in between the values for pure POPC and DPPC. Apparently, mixing of unsaturated and saturated lipids gives rise to a defect constant that is a (weighted) average of the π values for pure membranes, see the Discussion section and the SI, consistent with the idea that defect formation is a local phenomenon.

As before, the distributions obtained with Packmem’s CM and our TM overlap in all cases within reasonable error margins. Only for larger defects, associated with the lowest probability of sampling, can distributions be seen to deviate, albeit only slightly. In spite of this, fitted π^TM^ and π^CM^ are equivalent, signaling that this mismatch is insignificant in the description of the defect characteristics, i.e. the descriptor π. We thus conclude that our protocol reproduces PackMem results for all considered lipid compositions and only report results of our TM protocol from here onward. For completeness, reference PackMem values are reported in the SI for all flat bilayers.

**Figure 4:**
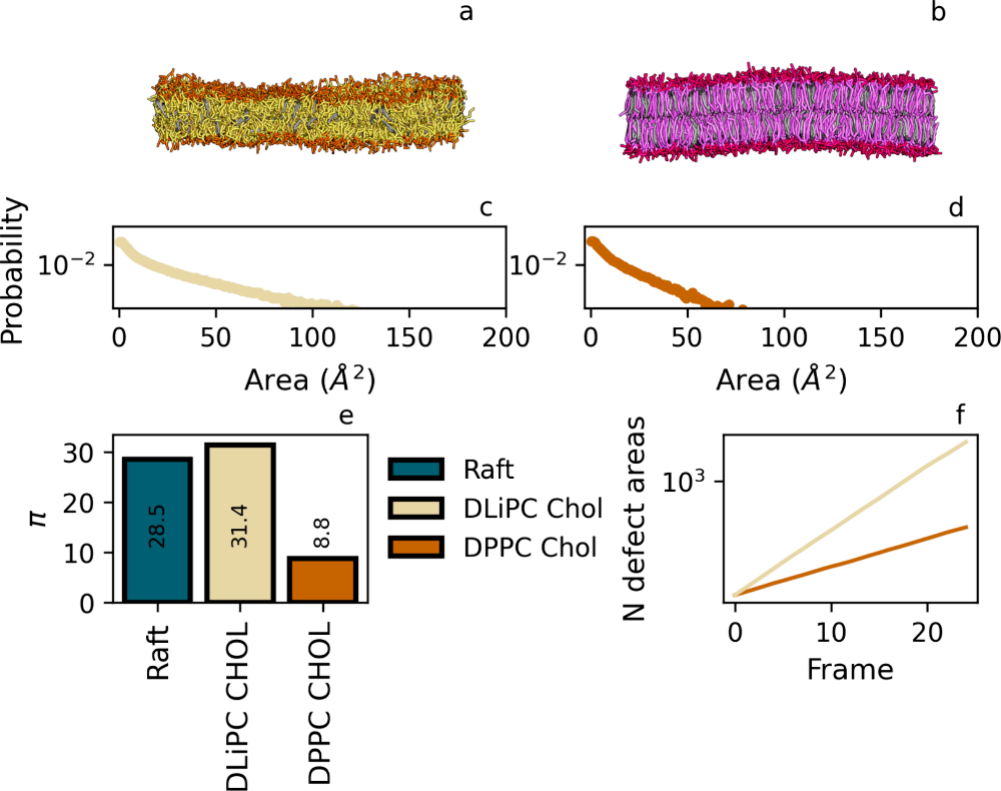
Defect properties of the raft and nonraft domains, simulated separately with lipid fractions extracted from the raft membrane. Panel a: fluid-like DLiPC/cholesterol mixture (85:15), with defect area distribution shown in panel c. Panel b: gel-like DPPC/cholesterol mixture (58:42), with defect area distribution shown in panel d. Panel e: π obtained by fitting, including the value obtained for the combined (raft) membrane. Panel f: total number of defects identified in each membrane over an increasing number of frames (yellow: DLiPC/cholesterol, orange: DPPC/cholesterol). Straight lines indicate that the number of defects per frame is constant for both membranes.

The finding of near-zero contact fractions for the raft membrane, see Figure 3, confirms the formation of distinct lipid domains, with a DPPC-cholesterol mixture forming one (raft) phase and DLiPC-cholesterol forming another. Since the choice for a single defect constant π for an entire membrane brings along the issue of representability when dealing with lateral separation in distinct domains, we additionally consider setups for the two individual phases. Figure 4 shows results for a DPPC:CHOL (58:42) and a DLiPC:CHOL (85:15) membrane, i.e. with fractions that have been matched to the composition in the two individual domains. Determining π for these two individual membranes illustrates that individual domains in the raft membrane indeed feature substantially different defect characteristics. In particular, the π for the membrane containing flexible DLiPC is quite high, with a value that is comparable to the π for the combined (raft) membrane, whereas the π for the membrane containing fully saturated DPPC is substantially reduced. Comparing these values, we may conclude that the overall defect properties of the raft membrane are dominated by the contribution of the DLiPC:CHOL domain. This is understandable, since this domain is more fluid-like, with lipids being less densely packed. In the other domain, i.e. formed by DPPC:CHOL, the lipid ordering is enhanced, resulting in defects that are clearly sparser and smaller, on average. This result already shows that the overall order has a profound effect on the defect characteristics and defect constant π. For this reason, we further concentrate on the role of lipid order, focusing on a pure DPPC membrane in the gel and liquid phase.

### The Role of Lipid Order

A general but lesser known challenge in coarse-grained modeling is that the formal map from the resolved to coarser molecular representation is valid only for the thermodynamic state for which this map was developed. While this is usually of no direct concern, for instance when one considers setups that stay relatively ’close’ to this reference state, it can in some cases pose a more serious challenge. For instance, a significantly reduced phase transition temperature was observed for a pure DPPC membrane in the CG Martini representation, which brings the simulated DPPC membrane in the liquid disordered state instead of the experimentally observed gel state at physiological temperature.^32^ Care should therefore be taken if lipid order within the membrane matters for the phenomena that one is interested in, for instance, when simulating peptide/protein binding at the CG level.

**Figure 5:**
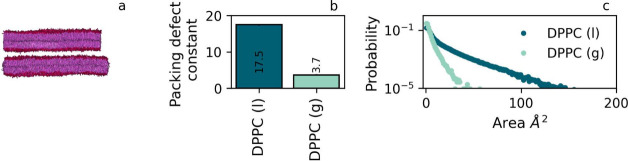
Defect properties for pure DPPC membranes in the gel and liquid disordered (liquid) phase. Panel a: snapshot of both membranes (upper: gel, lower: liquid). Panel b: π for the two membranes, corresponding to 17.5 ± 0.2 for DPPC (l) and 3.7 ± 0.1 for DPPC (g). Panel c: defect area distributions for both membranes.

Simulating a pure DPPC membrane at 285 K, i.e. below the Martini transition temperature of ≈290 K,^32,33^ brings it into the gel phase. We note that long equilibration times at decreased temperatures can be an issue in the preparation of such membranes. For the liquid disordered counterpart, we consider the largest pure DPPC membrane in Figure 2, which was simulated at 303 K, i.e. above the transition temperature in Martini. In agreement with the findings for the raft membrane in the previous subsection, we observe vastly different distributions of defect sizes for the two phases, see Figure 5. In particular, the ‘average’ defect size π in the gel phase is significantly reduced compared to that in the liquid (disordered) phase of a membrane of the same composition. These results confirm that lipid order indeed plays a key role in the defect characteristics and that increased lipid order gives rise to significant defect size reduction.

### Local Curvature

Next, we go beyond the applicability limits of PackMem and employ our protocol to membranes featuring distinct varying curvature along the membrane perimeter. Constant curvature that is present in perfectly cylindrical and spherical membranes has been previously analyzed using PackMem and was shown to give rise to a linear increase of the defect constant with curvature.^12^ Applying our protocol to two one-component liquid DPPC membranes of constant curvature, i.e. a cylinder and a sphere, confirms this finding, see the SI. Yet, many membranes in nature adopt much more complex shapes, featuring curvature that changes substantially with the position along the membrane. Such spatially varying curvature does not only arise during membrane remodeling, for instance in the formation of fusion stalks and tethers, but persistent local curvature is also present in various organelles and anticipated to play a key role in molecular recognition.^3,34^ Curvature-induced changes in the lipid packing can even affect the gel-to-liquid transition temperature, as shown by isothermal titration calorimetry experiments.^35^ In order to systematically analyze the role of the next factor, curvature, we first concentrate on buckled membranes of pure DPPC, both in the liquid and gel phase, containing well-defined domains of negative (J_–_), positive (J_+_), and zero (J_0_) curvature.

**Figure 6:**
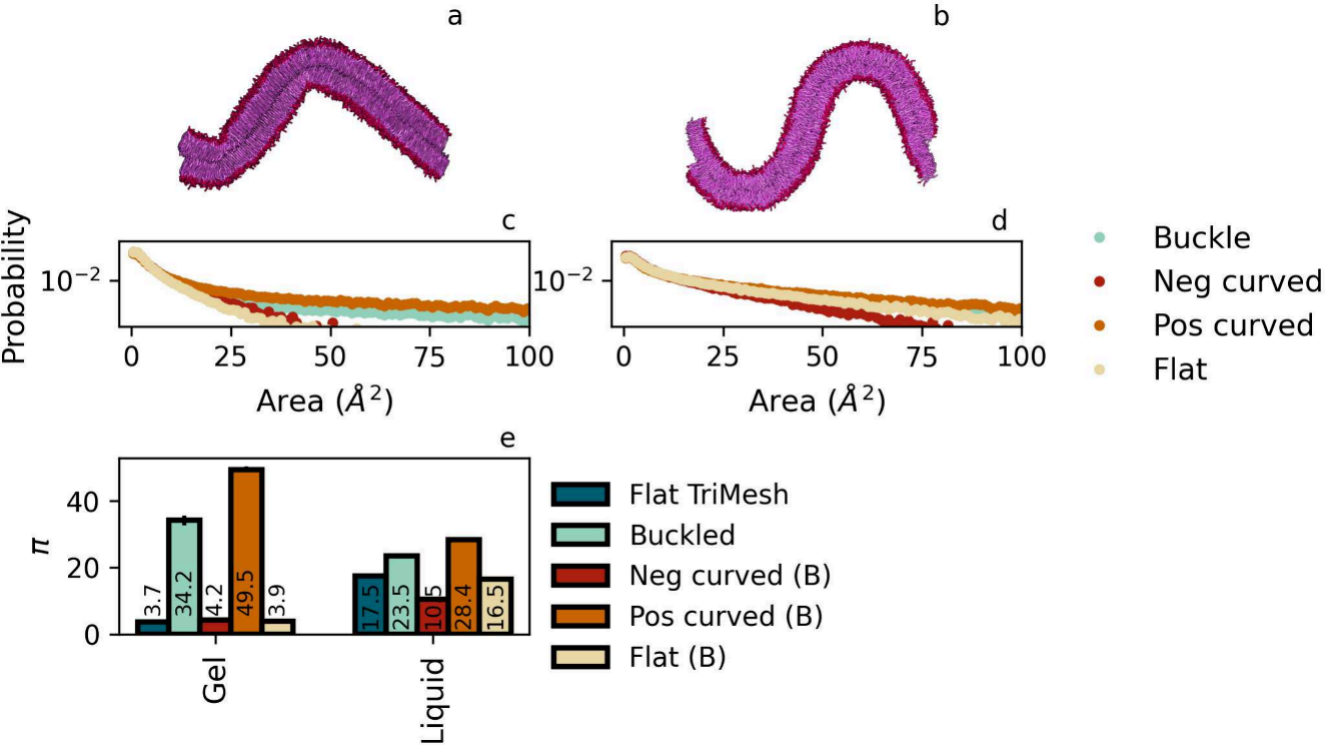
Defect analysis for membranes containing domains of positive and negative curvature. These buckled membranes are obtained by applying a constant strain to a flat membrane. Panel a: gel phase, and panel b: liquid phase. Panels c and d show the defect area probability of the corresponding buckles, as well as separate contributions stemming from the curved and flat domains. Panel e: defect constants π for the total membrane as well as positive, negative, and not curved parts of the buckled membranes. The limited size of domains with nonzero curvature has a notable effect on the quality of the fit.

The first surprise is that the order of the overall defect constants for the buckled membranes of DPPC gel and liquid phases, π_*buckle*_^*liquid*^ = 23.5 and π_*buckle*_^*gel*^ = 34.2, see Figure 6, is reversed compared to the values for flat membranes, where π_*flat*_^*liquid*^ = 17.5 and π_*flat*_^*gel*^ = 3.7. Moreover, the curvature appears to play only a marginal role in the liquid phase, increasing the average defect size only slightly. In the gel phase, on the other hand, curvature gives rise to a more than 8-fold increase of overall defect constant.

Differentiating for the role of local curvature, by splitting the buckle into three domains of distinct signature and fitting π from the defect distributions in these separate domains, see again Figure 6, tells a different story. One particularly finds that the defect characteristics of both membranes are dominated by contributions of the positively curved domains. The observation for the defect constants for the flat domain in the buckle, i.e. *π*_*J*_0__, are equivalent to the respective values for flat membranes π_*flat*_ again signifies that defect constants may be seen as a very local property. The small mismatch can be attributed to the reduced patch size of the three buckle domains considered. We conclude that, in the liquid phase, the role of curvature is in agreement with the (weak) linear relation observed previously for membranes with constant curvature, with positive curvature leading to a slightly increased π_*J*_+__ and negative curvature leading to a slightly reduced π_*J*_–__. For the gel phase, however, the situation is quite different. Here, positive curvature gives rise to a nonlinear increase of the defect numbers and sizes, even to the point where the average defect size is larger than that in any domain in the curved fluid membrane. We thus conclude that curvature will be a key factor in defect-driven phenomena for membranes that reside in the gel phase.

Experimental support for this conclusion is provided by a recent study of amyloid-β (Aβ) proteins.^36^ By systematically changing the membrane conditions, the authors demonstrated that Aβ binding is sensitive to curvature and that binding is restricted to electrostatically neutral lipid membranes in the gel phase and anionic lipid membranes in the liquid-crystalline phase.^36^ In particular, bound Aβ was found exclusively at the highly curved edge domains of 30 nm polyhedral vesicles in the gel phase. This finding shows that the defect dimension required for granting Aβ access to the hydrophobic membrane interior is indeed restricted to the gel edge, as rationalized by our computational results.

### Other Membrane Geometries

We have thus far considered fairly simple geometries to systematically evaluate key contributions to lipid packing defects. As a final application of our protocol, we examine the defect characteristics of two biologically relevant structures that feature distinct local curvature and thereby fall outside the application range of the existing method. The first one is a delivery system for hydrophobic drugs, i.e. a lipid nanoparticle (LN), that overall adopts a spherical geometry but easily deforms. The second is a complicated junction structure that represents a stalk domain formed during liposome fusion. Both membranes are composed of a mixture of a standard phospholipid and diacylglycerol (DAG) that prefers negative curvature. The insight gained by simulation of these membranes for particular applications/properties has been discussed elsewhere.^37^ Here, we report only their defect properties.

**Figure 7:**
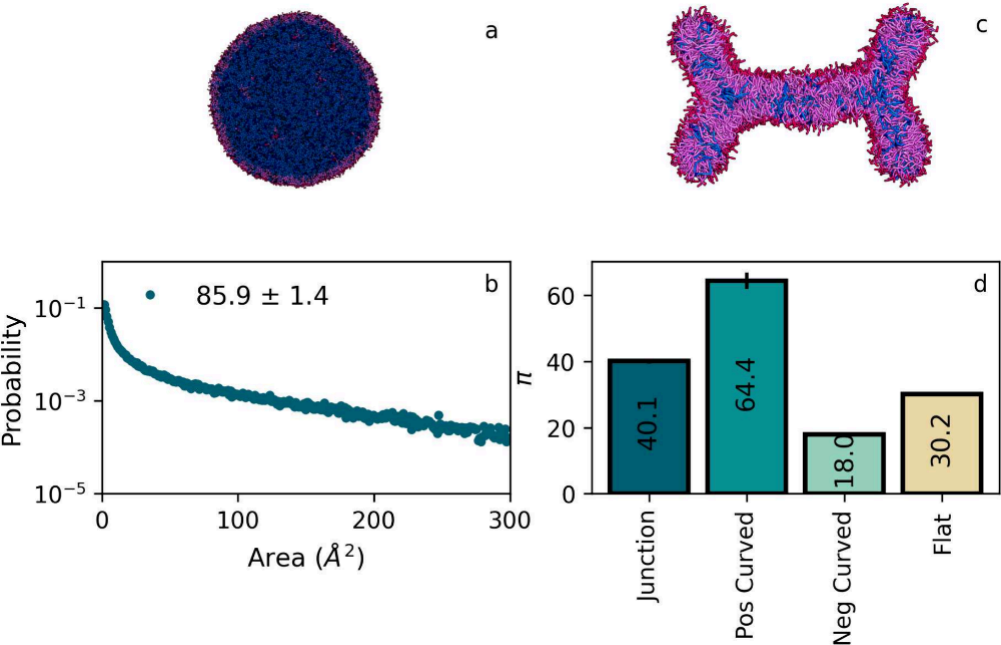
Defect analysis for irregular membrane geometries that may be formed in nature, such as a lipid nanoparticle (LN) and junctioned membranes, that are related to intermediate stalk geometries in fusion. Panel a: snapshot of the LN, and panel b: the defect area distribution in the LN monolayer, for which π = 85.9 ± 1.4 Å^2^ is fitted. Panel c: snapshot of the junction membrane, in which DPPC lipids are displayed as a red headgroup and two purple tails, while DAG lipids are shown as blue. Panel d: π as obtained by fitting defect distribution of the entire junction membrane, as well as separated domains of specific curvature. We used the same protocol as that for the buckles.

First, we note that although the LN, see Figure 7, appears to be spherical, it does essentially require a nonspherical mesh to determine a proper defect constant π. The LN becomes nonspherical owing to a rather low interfacial tension, and a careful check using spherical coordinates showed us that these deformations are well beyond the limits of the PackMem mapping protocol, which allows only a very modest deviation of perfect sphericity. It should be understood that deriving proper π-values for arbitrarily curved systems like the LN is of importance. For instance, a recently developed technique for the computational design of sensor peptides requires π-values for a curved target membrane as an input,^37^ since it takes the alternative route of applying mechanical stress to a flat membrane to tune defect characteristics toward reference values, for reasons of computational efficiency.^2^ Without our protocol, such reference values cannot be determined. As the area of these deformations is overall quite small and they share the curvature sign, we only consider the overall defect constant. This value is rather high, namely π_LN_ = 85.9 ± 1.4, reflecting the combined effect of curvature and particular DPPC and DAG packing.

Next, we concentrate on the junction, and we clearly observe membrane domains of distinct curvature, meaning that a packing defect constant for the entire membrane is irrelevant for the understanding of defect-mediated phenomena. As before, we use relevant threshold values to couple lipid pools to the signature of curvature and split the membrane into three representative curvature domains, see the Supporting Information for details. Again, a closer look at the curvature-differentiated distributions, see Figure 7, suggests that larger defects may be undersampled due to a limited size of the curved membrane domains. Yet, fitting these distributions provides π_*J*_+ = 64.4, π_*J*_– = 18.0, and π_*J*_0__ = 30.2, and an overall defect constant of π = 40.1, indicating that the average defect size is again largest in domains of positive curvature, followed by flat domains. In this particular case, domains of positive curvature are absent in an actual fusion stalk, so one may conclude that defect mediated phenomena are most probable along the flat connection region.

## Discussion

Our results clearly demonstrate that two factors, lipid order and curvature, provide very distinct contributions to the standard descriptor for defects, i.e. the defect constant π, for an entire membrane. They also show that these contributions are often hidden in this descriptor since a single value for the entire membrane primarily reflects defect properties of curved domains or, for a flat membrane, the most flexible lipid component. This may pose a challenge when considering biological membranes, which generally have a very diverse lipid content and display emergent physicochemical properties via lipids that collectively regulate membrane function. An example is the formation of lipid membrane domains that are anticipated to play a role in protein binding, which are enriched in particular lipids and often of distinct order. One may thus conclude that, even for an (approximately) flat membrane, a single defect constant π is insufficient for capturing this important functional detail. Moreover, the membrane morphology is controlled by several thermodynamic forces and usually inherently features instabilities that may be released via the formation of local or global curvature with very particular defect characteristics. Overall, this shows that there is a clear need for differentiating between these factors when one is interested in understanding the role of the defect structure in phenomena that take place at the membranes interface. Next, we discuss a number of additional observations.

### Traditional Membrane Descriptors

Composition is the third variable that is seen to regulate the defect constant π, but it is still rather unspecified how the lipid type/chemistry correlates with the defect characteristics, even when membranes feature no curvature and are in the usual liquid disordered phase. Our result for POPC and DPPC membranes show that π_POPC_ > π_DPPC_ for flat fluid membranes. The denser packing of the rather rigid DPPC compared to that of more flexible POPC at the same temperature *T* is a factor. As packing, and the room for ‘wiggling’ within a lipid ensemble, is also reflected in standard membrane descriptors, the following question arises: is there a hidden correlation to one or more traditional membrane descriptors that allows us to simply estimate π from existing data?

**Figure 8:**
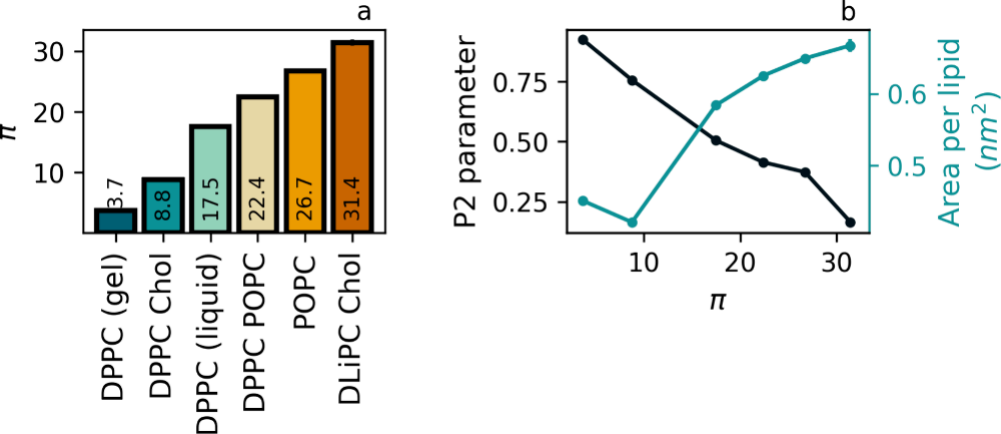
(a) Values of the defect constant π obtained for the membranes considered in this paper, ordered by increasing magnitude. (b) P_2_ orientational order parameter (black) and average area per lipid (blue), determined using standard routines in Gromacs, versus π. We note that all membranes were simulated using the latest version Martini 3, except for membranes containing cholesterol, which were simulated using Martini 2, see discussion on the role of the force field. The reason for this choice is the current lack of a proper cholesterol parametrization in Martini 3. Since the number of data points is restricted, we did not perform regression analysis.

Figure 8a shows that π for fully saturated DPPC indeed increases with increasing disorder: from a gel, a gel with added cholesterol, a liquid, and a liquid mixture with POPC. This makes sense, since adding cholesterol to a one-component gel membrane is known to enhance fluidity, and POPC has one unsaturated bond. DLiPC is an even more unsaturated counterpart to fully saturated DPPC, containing four unsaturated bonds versus only one for POPC. Adding 15% cholesterol will however only very mildly perturb the lipid order, as seen from a tiny increase of the orientational order parameter P_2_ in an earlier study.^38^ It thus makes sense that the DLiPC mixture possesses the largest ‘wiggle’ space of all PC lipids considered in this study. Panel b of Figure 8 shows that the defect constants π for the six considered setups in this study indeed correlate quite well with P_2_ calculated for the most flexible tail in the lipid, containing unsaturated bonds, even for lipid mixtures. One expects that standard membrane descriptors like the area per lipid (APL) should also correlate with π, as it relates to the average space that each lipid occupies, and indeed it appears to behave like this. We note that the membranes containing cholesterol have been simulated using Martini 2 instead of the latest Martini 3 of all other membranes in Figure 8, because of a missing parametrization for this component in Martini 3.

We should, however, be aware that all considered lipids here share a PC headgroup, except for cholesterol, and that the volume of this headgroup plays a role in lipid packing. For this reason, we have also considered data from the literature; see the combined plot in the Supporting Information. A complicating factor for straightforward analysis is that values for the defect constant π and standard descriptors like P_2_ and APL are seldom published in combination. Moreover, it is not easy to quantify the role of different force fields, temperatures, etc. that is needed for combining data from different sources. This limited exercise, however, suggests that it could indeed be possible to reliably estimate the defect constant from standard membrane descriptors, which would open up an avenue for machine learning. Further study is required but might be very beneficial.

### Limited Domain Sizes

While the increase of defect sizes by curvature is substantial in the gel phase, curvature is generally not evenly distributed over the entire lipid structure in this case. In particular, below the phase transition temperature, DPPC/cholesterol mixtures have experimentally been observed to form faceted vesicles in order to accommodate the large penalty for bending. They thus combine large, flat facets with a few curved edges. Consequently, *functional* defects, i.e. defects that exceed a certain minimal size, are likely only present at these edges in the gel phase, and proteins that depend on them for binding need to be abundant in the solvent phase or require ample time to diffuse to these edge domains. To facilitate future experimental validation of this effect, it thus makes sense to estimate the fraction of binding area on such vesicles; see the Supporting Information. Although depending on particular shape and radius, a general observation is that the surface area accessible for binding to a gel DPPC vesicle would generally be well below 10% of the total vesicle area, see Supporting Information, assuming that all flat vesicle domains are nonbinding.

A similar issue plays a role in computational defect analysis of curved structures, i.e. for the buckled and junction membranes. After all, the considered CG membranes are, albeit much larger than in fully atomistic simulations, still rather modest in size, which renders the area occupied by curved domains in the simulations even more restricted. From the results for flat membranes, one may conclude that fitting a defect distribution for a 10 × 10 nm patch size gives rise to an accurate defect constant π, i.e. within a few percent of a ‘converged’ value for infinitely large patches, despite a reduced signal-to-noise ratio. Particularly not only for the junction but also for the buckle membrane, the total area of the curved domains falls short of this 100 nm^2^, meaning that we consider cases for which the role of sampling issues has not been tested. On the other hand, simulating much larger curved membranes or much longer time traces to investigate the precision has significant consequences for the computational costs. Apart from the quality of our fit benefiting from the careful statistical analysis that we introduced, we argue that the identification of curvature- and phase-related trends will not change with enhanced sampling, albeit that estimates will converge to sampling-invariant values. This conclusion is supported by the rather comparable π values identified for flat membranes and flat parts in the buckles. Quantitatively, we performed extended sampling for one selected setup, and we observed an increase in π_*J*_+__ (data not shown). This suggests that fitted defect constants for curved domains may be somewhat underestimated compared to actual values for these geometries.

### Sensitivity to the Force Field

It should be understood that calculated defect constants π are essentially force field (FF) dependent, a finding that can be understood in terms of the particular projected particle sizes in different molecular representations. While one may anticipate that a simple correction relation exists, momentarily this relation is unknown and obstructs the direct comparison of the π-value obtained using different FFs. Previously, significant mismatch was reported for the defect analysis of one-component membranes composed of DMPC, POPC, DOPC or DPPC lipids and for DOPC/DOG mixed membranes simulated using two different atomistic FFs, CHARMM36 and Berger.^13^ Whereas π values were found to be ill-defined, in the sense that they vary significantly with the considered FF, an effect that was attributed to the all-atom versus united-atom nature of the FF, the good news is that the *relative* values or trends appear to be conserved. In this study, we have complemented this FF dependency by the observation that π values are distinct up to a few percent even when considering different instances of the same system and the same FF, owing to the finite sampling in practice and fitting uncertainties.

The π values reported in this study are, as a whole, elevated compared to values identified for the same membrane compositions in the literature. Yet, also in this case, we mainly employed the recently introduced Martini 3 FF instead of the standard Martini 2 FF of older studies. Just like in the all-atom case discussed above, the different values can be attributed to dealing with detail, in our case, the Van der Waals radii, that is used in defect assignment, or the particular distance to *T*_*c*_ in the considered AA-to-CG map. It should, however, be understood that π values determined using our protocol and Packmem for the same FF, the same temperature, and membrane composition have been found to compare very well in this study.

### Defect Constants for Lipid Mixtures

The finding that lipid packing defects are local and noninteracting^3^ suggests that the defect constant for complex membrane simply follows from simpler ones via a superposition principle. Defect constants for one-component membranes would then suffice as a basis for all of the π. The single exponential fit that is identified for mixed membranes supports this idea. Yet, while this relation is easy to grasp for membranes that phase separate into distinct domains, like the raft membrane, it is less obvious when the lipids in the membrane are fully mixed. To test our ansatz, we have derived a relation for the most basic setup: a membrane that is a mixture of two different lipid types or of one lipid type in two different phases; see the Supporting Information. The defect constant π_3_ for the mixture is given by two equivalent relations as
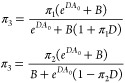
2where π_1_ and π_2_ are the defect constants for the pure systems, and *B* and *D* are given in the SI. The value of *A*_0_ can be freely chosen within the interval where the exponential fit is valid. We have considered *A*_0_ = 50 Å^2^ for the remainder.

We may test expression 2 for its predictive value. Considering the mixed membrane of DPPC and POPC, we may estimate π_3_ from the values for the one-component DPPC and POPC membranes. For the raft membrane, which is a three-component system consisting of a mixture of two lipids and cholesterol, the two individual lipid/cholesterol membranes may serve as a base. Although the buckled membrane is a more genuine three-component system in terms of curvature, we may consider the possibility of only the flat and the positively curved domains providing input values. While a three-component version of (2) is in principle possible, even as a combination of two two-component systems, it will illustrate whether domains with negative curvature significantly perturb the π for the entire membrane. Table 2 compares the exponential fit results for the mixed membrane and predictions using (2). Overall, predicted values match the actual ones quite well, within a small percent error margin. For the buckled membrane, the predictions on the basis of two components match quite well for the liquid membrane, while the prediction is quite far off for the gel phase. Clearly, such a simple relation does not necessarily hold when curvature is involved for a gel membrane, in line with the other finding of nonlinearity, and additional investigation is required. While the predictions for fluid membranes are encouraging, also in that case more analysis is desired, for instance in selecting a proper value of *A*_0_, before we can draw more firm conclusions about the validity of this approach.

**Table 2:** Superposition Enables Us to Compute Values for Mixtures from the Base Values for Pure Systems: Here, π Values for Three Different Membranes Are Predicted and Compared to Fitted Values: 1) a Membrane Consisting of DPPC and POPC (1:1), 2) a Raft Membrane Consisting of a DPPC with a Cholesterol-Rich Region (58:42) and a DIPC with a Cholesterol Deficient Region (85:15), and 3) a One-Component Buckled Membrane in the Gas (g) and Liquid (l) Phase from the Values for the Flat and Curved Regions^a^

Membrane	π	Membrane	π
DPPC (l)	17.5 ± 0.2	DPPC/cholesterol	8.8 ± 0.2
POPC (l)	26.7 ± 0.3	DLiPC/cholesterol	31.4 ± 0.5
DPPC/POPC	22.4 ± 0.2	Raft	28.5 ± 0.3
Predicted	22.9	Predicted	27.2
Flat (g)	3.9 ± 0.1	Flat (l)	16.5 ± 0.3
Curved (g)	49.5 ± 0.9	Curved (l)	28.5 ± 0.5
Buckle (g)	34.2 ± 1.5	Buckle (l) (l)	23.5 ± 0.4
Predicted (g)	49.3	Predicted (l)	22.7

a In the latter case, we disregarded negative curvature in the prediction.

We conclude with the remark that if we combine the predictions for π from standard membrane descriptors like APL, see the earlier paragraph, with the superposition principle for mixed systems, we would obtain an easy route for the determination of defect constants for a large set of (mixed) fluid lipid membranes. In particular, standard descriptors for a range of flat lipid membranes are available from the literature, originating from both computational and experimental work. After extensive testing of well-chosen predictions to actual fitted π, this would provide a good training set for machine learning at minimal computational costs. The role of curvature, which is more significant as illustrated, would require additional investigation, but the same principles would apply.

### Defect Definition

We did not make a distinction between deep and shallow in the current paper. First, we should acknowledge the term ‘defect’ does not provide a precise description of a relevant phenomenon. It signifies regions where the hydrophobic core of the membrane becomes exposed to environmental proteins that contain hydrophobic domains that actively seek such regions. Therefore, the previously mentioned surface accessible hydrophobic areas (SAHAs) or exposed hydrophobic core at the interface may be a more accurate description. Second, it is worth noting that a subtle difference exists between different defect types and also that additional categories have been considered.^14^ In particular, shallow defects refer to surface-level irregularities arising from local disruptions in the structuring of lipid headgroups, while deep defects involve disruptions occurring deeper within the bilayer, affecting the lipid tails. It has been proposed that smaller amino acids bind more easily to shallow defects and larger, bulkier ones bind more easily to deeper defects. However, this distinction is not relevant in the current context. The methodology developed and employed here has the built-in capacity to distinguish between these types of defects if desired. In such a case, we should use a depth of 4.5 Å, which was determined based on reproduction of PackMem results.

### Statistical Methodology

The challenge of fitting a defect area distribution obtained via simulation to a known function is a standard issue in (linear) regression but has not been discussed in that context before.^12,13^ Previously, it was however recognized that the selection of a fitting range plays a crucial role in the determination of π. Analysis of this dependency resulted in a selection of fixed lower bound of 15 Å^2^ and often also an upper bound.^13^ For membranes containing very few and small defects, like the well-ordered (gel) membranes considered in this study, this standard lower bound of PackMem may lead to a serious fitting challenge, as most of the data have to be discarded.

In the Supporting Information, we clarify that the range corresponding to the smallest defects indeed should be disregarded, as these data represent local order in the form of excluded volume for a system of hard spheres rather than information about actual defects. Meanwhile, the highest range should be truncated because of a low signal-to-noise ratio. In particular, the exponential nature shows that, as defects increase in size, their likelihood to be sampled drops exponentially. The negligible variation in the defect constants that were calculated for three DPPC lipid patches of different sizes exemplifies that the selection of a proper fitting range is a good remedy. Here, we have followed the principles of regression for each individual case, which state that the verification of the fit quality is essential for ensuring reliable outcomes; see the Methods section and the SI.

### Other Application of the Mapping Scheme

Recently, Pezeshkian et al. developed a TS2CG backmapping procedure from a triangulated surface to a molecular CG representation that is complementary to ours.^39^ While TS2CG enables direct structural input from the efficient elastic continuum theory (ECT), which is based on a Helfrich free energy and takes only effective membrane parameters such as bending rigidity and surface tension as input, they illustrated TS2CG by backmapping a mitochondrial structure, triangulated from an electron microscopy (EM) image, and showing that filled in molecular structure by backmapping is stable under (short) CG simulation. Many of the established particle-based CG representations for lipid membranes, which range from highly resolved united atoms^4^ to a rather coarse three bead model developed by Cooke and Deserno,^40^ are bridged by mappings, with the role of (explicit or implicit) solvent and the conservation of selected thermodynamic properties by coarsening giving rise to distinct procedures. Particularly in cases where compositional heterogeneity and molecular resolution are insignificant for studying a phenomenon of interest, for instance to investigate the remodeling kinetics of giant unilamellar vesicles (GUVs) induced by internal or external factors, continuum approaches like molecular field theory (MFT)^41^ and ECT^42^ may be a viable option.

Multiscale approaches based on consecutive forward and backmappings between different resolutions are increasingly employed to exploit equivalence for computational efficiency and to resolve issues associated with simulating only at a single resolution. General examples are the slow evolution at the AA scale and discarded molecular interaction detail in CG. The required equivalence, however, poses a genuine challenge when one wants to apply the same consecutive procedure between the molecule-resolved and continuum domains. After all, for lipid mixtures, molecular detail is generally responsive to or even responsible for membrane modulation at a larger scale, for instance, for relieving local stresses, and the continuum treatment should reflect this property. For MFT or self-consistent field theory (SCFT), under which name it is popular in the polymer community, this can be most effectively resolved by hybridization. In recent years, few hybrid approaches have been formulated that combine molecular (AA or CG) detail and bonded interactions with a SCFT-inspired evaluation of nonbonded interactions in terms of continuum fields.^43,44^ While the true challenge in forward mapping CG results to elastic theory lies in assuring equivalence of the ECT treatment that should follow, a proper forward map does not even exist, see the discussion in the study of the TS2CG backmap.^39^ Here, we have introduced this desired forward CG2TS map but for an entirely different purpose.

## Conclusion

We have developed a CG2TS protocol that can translate molecular membrane simulation results into triangulated continuum surfaces that represent membrane leaflets. This protocol is subsequently employed to analyze the role of several key membrane properties in the nature of lipid packing defects, including local curvature, lipid packing, and lipid mixing. As defects are known to play a key role in the binding of peptides, proteins, and nanoparticles to biomembranes, quantifying the defects characteristics represents a necessary step toward a better understanding of membrane function. The applicability of this protocol to membranes of any shape and composition, even in the case of irregular geometries that are abundant in biology, constitutes a major step forward in the computational research of specific molecule-membrane interactions.

Our study has identified a strong correlation between lipid tail order and the defect constant π, which is a descriptor for the average defect size. In particular, an increased lipid tail order gives rise to a reduced π. As a result, the π for a one-component membrane is very sensitive to the gel-to-liquid phase transition temperature *T*_*c*_ associated with the constituting lipid. For *T*_*c*_, i.e. when a membrane is in a liquid disordered phase, π is high. Yet, even within this phase, π is sensitive to the relative tail order. For more flexible lipids, which is equivalent to lipids with a lower *T*_*c*_, π is larger at a fixed temperature *T* than that for more rigid lipids or lipids with a higher *T*_*c*_. When a membrane has passed from the liquid-disordered (liquid) into the gel phase, i.e. *T* < *T*_*c*_, π drops to a much reduced value. This value may be so low that the associated defects even inhibit binding. The observation that lipid order is already captured by standard membrane descriptors, such as not only the orientational order parameter P_2_ but also the area per lipid (APL), suggests that π could also be expressed in terms of membrane descriptors that have already been determined for many setups. More study is needed to, however, determine the exact relation.

Previous studies for tubes and vesicles have indicated that curvature, which is known to be an important factor in biology, generally increases π compared to their flat counterpart. It should be noted that membrane curvature can only play a role in the defect size since the pivotal plane, i.e. the surface at which the area strain vanishes,^45^ differs from the plane at which the defects of interest arise. Basic geometrical analysis suggests that the closer this pivotal plane lies to the mid plane, or the further away from the plane of defects, the stronger the defect size will increase with curvature. For the gel phase, one thus expects a stronger effect. Evaluating the defect characteristics for membranes that curve under applied stress, i.e. a buckled membrane, and a mixed membrane featuring an irregular geometry with local curvature, we find that local curvature does not necessarily increase the π for the entire membrane. In positively curved domains, however, the π increases, while the π decreases in negatively curved domains. The finding that this trend is the same in both one- and two-component membranes, with the second lipid preferring negative curvature, suggests that it has a more general validity. An interesting finding is the unexpected interplay of the curvature and order. We particularly find that, in domains of high positive curvature, a lipid membrane in the gel phase can feature larger (average) packing defects than the maximum π of the same membrane in the liquid phase. An implication of this finding is that gel membranes may be susceptible to binding, despite the close packing of the lipids. However, the total domain size available for binding is considerably reduced.

This result illustrates once more why a defect constant π for an entire membrane cannot adequately describe defect features in membranes that exhibit variations in tail ordering, curvature, or composition along the membrane perimeter. It also generates first insight into the importance of membrane detail in binding and of getting this detail right in a computational setup when studying binding properties. An example is the majority-DPPC lung membrane, which features both different phases and high curvatures with a yet largely unspecified role.^46^ As an additional feature, the CG2TS protocol can also be exploited to map CG results into a continuum description for the advantage of enhancing sampling in the phase space. Application of CG2TS for this purpose is left to a future study.

## Supporting Information Available

Implementation of the CG2TS projection. Domain assignment based on curvature and contact fraction calculation. Considerations about curvature-π linearity in the liquid state and relative curvature exposure in the gel phase. Equations for π superposition for mixed systems and correlations with existing membrane descriptors from literature. The fitting procedure and data (PDF)

## References

[ref1] Hoffmann L.; Carenza L.; Giomi L. Tuneable defect-curvature coupling and topological transitions in active shells. Soft Matter 2023, 19, 3423–3435. 10.1039/D2SM01370C. 37129899

[ref2] van Hilten N.; Steffen Stroh K.; Risselada H. Efficient Quantification of Lipid Packing Defect Sensing by Amphipathic Peptides: Comparing Martini 2 and 3 with CHARMM36. J. Chem. Theory Comput. 2022, 18, 4503–4514. 10.1021/acs.jctc.2c00222. 35709386 PMC9281404

[ref3] Cui H.; Lyman E.; Voth G. Mechanism of membrane curvature sensing by amphipathic helix containing proteins. Biophys. J. 2011, 100, 1271–1279. 10.1016/j.bpj.2011.01.036. 21354400 PMC3043213

[ref4] Allen W. J.; Lemkul J. A.; Bevan D. R. Gridmat-MD: A Grid-Based Membrane Analysis Tool for Use with Molecular Dynamics. J. Comput. Chem. 2009, 30, 1952–1958. 10.1002/jcc.21172. 19090582

[ref5] Lukat G.; Kruger J.; Sommer B. Apl@Voro: A Voronoi-Based Membrane Analysis Tool for Gromacs Trajectories. J. Chem. Inf. Model. 2013, 53, 2908–2925. 10.1021/ci400172g. 24175728

[ref6] Guixa-Gonzalez R.; Rodriguez-Espigares I.; Ramirez-Anguita J. M.; Carrio-Gaspar P.; Martinez-Seara H.; Giorgino T.; Selent J. Membplugin: Studying Membrane Complexity in Vmd. Bioinformatics 2014, 30, 1478–1480. 10.1093/bioinformatics/btu037. 24451625

[ref7] Carr M.; MacPhee C. E. Membrainy: A Smart, Unified Membrane Analysis Tool. Source Code Biol. Med. 2015, 10, 3 10.1186/s13029-015-0033-7. 26060507 PMC4460882

[ref8] Yesylevskyy S. O.; Ramseyer C. Determination of Mean and Gaussian Curvatures of Highly Curved Asymmetric Lipid Bilayers: The Case Study of the Influence of Cholesterol on the Membrane Shape. Phys. Chem. Chem. Phys. 2014, 16, 17052–17061. 10.1039/C4CP01544D. 25004951

[ref9] Buchoux S.; Fatslim A. Fast and Robust Software to Analyze Md Simulations of Membranes. Bioinformatics 2017, 33, 133–134. 10.1093/bioinformatics/btw563. 27578804

[ref10] Santos D.; Pontes F.; Lins R.; Coutinho K.; Soares T. SuAVE: A Tool for Analyzing Curvature-Dependent Properties in Chemical Interfaces. J. Chem. Inf. Model. 2020, 60, 473–484. 10.1021/acs.jcim.9b00569. 31508962

[ref11] Santos D.; Coutinho K.; Soares T. Surface Assessment via Grid Evaluation (SuAVE) for Every Surface Curvature and Cavity Shape. J. Chem. Inf. Model. 2022, 62, 4690 10.1021/acs.jcim.2c00673. 35946873 PMC9554907

[ref12] Vanni S.; Hirose H.; Barelli H.; Antonny B.; Gautier R. A sub-nanometre view of how membrane curvature and composition modulate lipid packing and protein recruitment. Nat. Commun. 2014, 5, 4916 10.1038/ncomms5916. 25222832

[ref13] Gautier R.; Bacle A.; Tiberti M.; Fuchs P.; Vanni S.; Antonny B. PackMem: AVersatile Tool to Compute and Visualize Interfacial Packing Defects in Lipid Bilayers. Biophys. J. 2018, 115, 436–444. 10.1016/j.bpj.2018.06.025. 30055754 PMC6084522

[ref14] Tripathy M.; Thangamani S.; Srivastava A. Three-Dimensional Packing Defects in Lipid Membrane as a Function of Membrane Order. J. Chem. Theory Comput. 2020, 16, 7800–7816. 10.1021/acs.jctc.0c00609. 33226805

[ref15] Grimmett G. In Percolation; Springer Berlin Heidelberg: Berlin, Heidelberg, 1999; pp 1–31.

[ref16] Gowers R.; Linke M.; Barnoud J.; Reddy T.; Melo M. N.; Seyler S.; Dotson D.; Domanski J.; Buchoux S.; Kenney I.; Beckstein O. MDAnalysis: A Python package for the rapid analysis of molecular dynamics simulations. Proceedings of the 15th Python in Science Conference; 2016; pp 98–105.

[ref17] Michaud-Agrawal N.; Denning E.; Woolf T.; Beckstein O. MDAnalysis: A Toolkit for the Analysis of Molecular Dynamics Simulations. J. Comput. Chem. 2011, 32, 2319–2327. 10.1002/jcc.21787. 21500218 PMC3144279

[ref18] Kazhdan M.; Bolitho M.; Hoppe H. Poisson Surface Reconstruction. Symposium on Geometry Processing; 2006; pp 1727–8384.

[ref19] Du Q.; Faber V.; Gunzburger M. Centroidal Voronoi Tessellations: Applications and Algorithms. SIAM Review 1999, 41, 637–676. 10.1137/S0036144599352836.

[ref20] James G.; Witten D.; Hastie T.; Tibshirani R.; Taylor J. An Introduction to Statistical Learning; Springer: 2023.

[ref21] Souza P.; Alessandri R.; Barnoud J.; Thallmair S.; Faustino I.; Grönewald F.; Patmanidis I.; Abdizadeh H.; Bruininks B.; Wassenaar T.; Kroon P.; Melcr J.; Nieto V.; Corradi V.; Khan H.; Domaaski J.; Javanainen M.; Martinez-Seara H.; Reuter N.; Best B.; Vattulainen R.; Monticelli I.; Periole L.; Tieleman X.; de Vries D. P.; Marrink A.; Martini S. Martini 3: a general purpose force field for coarse-grained molecular dynamics. Nat. Methods 2021, 18, 382–388. 10.1038/s41592-021-01098-3. 33782607 PMC12554258

[ref22] Marrink S.; Risselada H.; Yefimov S.; Tieleman D.; de Vries A. The MARTINI Force Field: Coarse Grained Model for Biomolecular Simulations. J. Phys. Chem. B 2007, 111, 7812–7824. 10.1021/jp071097f. 17569554

[ref23] Zoni V.; Khaddaj R.; Lukmantara I.; Shinoda W.; Yang H.; Schneiter R.; Vanni S. Seipin accumulates and traps diacylglycerols and triglycerides in its ring-like structure. Proc. Natl. Acad. Sci. U.S.A. 2021, 118, e2017205118 10.1073/pnas.2017205118. 33674387 PMC7958289

[ref24] Campomanes P.; Zoni V.; Vanni S. Local accumulation of diacylglycerol alters membrane properties nonlinearly due to its transbilayer activity. Communications Chemistry 2019, 2, 72 10.1038/s42004-019-0175-7.

[ref25] Thompson A.; Aktulga H.; Berger R.; Bolintineanu D.; Brown W.; Crozier P.; in ’t Veld P.; Kohlmeyer A.; Moore S.; Nguyen T.; Shan R.; Stevens M.; Tranchida J.; Trott C.; Plimpton S. LAMMPS - a flexible simulation tool for particle-based materials modeling at the atomic, meso, and continuum scales. Comput. Phys. Commun. 2022, 271, 108171 10.1016/j.cpc.2021.108171.

[ref26] Plimpton S. Fast Parallel Algorithms for Short-Range Molecular Dynamics. J. Comput. Phys. 1995, 117, 1–19. 10.1006/jcph.1995.1039.

[ref27] Jo S.; Kim T.; Iyer V.; Im W. CHARMM-GUI: A web-based graphical user interface for CHARMM. J. Comput. Chem. 2008, 29, 1859–1865. 10.1002/jcc.20945. 18351591

[ref28] Lee J.; Cheng X.; Swails J.; Yeom M.; Eastman P.; Lemkul J.; Wei S.; Buckner J.; Jeong J. C.; Qi Y.; Jo S.; Pande V.; Case D.; Brooks C. I.; MacKerell A. J.; Klauda J.; Im W. CHARMM-GUI Input Generator for NAMD, GROMACS, AMBER, OpenMM, and CHARMM/OpenMM Simulations Using the CHARMM36 Additive Force Field. J. Chem. Theory Comput. 2016, 12, 405–413. 10.1021/acs.jctc.5b00935. 26631602 PMC4712441

[ref29] Martinez L.; Andrade R.; Birgin E. G.; Martí-nez J. M. PACKMOL: A package for building initial configurations for molecular dynamics simulations. J. Comput. Chem. 2009, 30, 2157–2164. 10.1002/jcc.21224. 19229944

[ref30] Van der Veen J.; Kennelly J.; Wan S.; Vance J.; Vance D.; Jacobs R. The critical role of phosphatidylcholine and phosphatidylethanolamine metabolism in health and disease. BBA-Biomembranes 2017, 1859, 1558–1572. 10.1016/j.bbamem.2017.04.006. 28411170

[ref31] Liu Y.; de Vries A.; Pezeshkian W.; Marrink S. Capturing Membrane Phase Separation by Dual Resolution Molecular Dynamics Simulations. J. Chem. Theory Comput. 2021, 17, 5876–5884. 10.1021/acs.jctc.1c00151. 34165988 PMC8444333

[ref32] Jaschonek S.; Cascella M.; Gauss J.; Diezemann G.; Milano G. Intramolecular structural parameters are key modulators of the gel-liquid transition in coarse grained simulations of DPPC and DOPC lipid bilayers. Biochem. Biophys. Res. Commun. 2018, 498, 327–333. 10.1016/j.bbrc.2017.10.132. 29101041

[ref33] Risselada H.; Marrink S. The freezing process of small lipid vesicles at molecular resolution. Soft Matter 2009, 5, 4531–4541. 10.1039/b913210d.

[ref34] Has C.; Sivadas P.; Lal Das S. Insights into Membrane Curvature Sensing and Membrane Remodeling by Intrinsically Disordered Proteins and Protein Regions. J. Membr. Biol. 2022, 255, 237–259. 10.1007/s00232-022-00237-x. 35451616 PMC9028910

[ref35] Yokoyama H.; Ikedai K.; Wakabayashi M.; Ishihama Y.; Nakano M. Effects of Lipid Membrane Curvature on Lipid Packing State Evaluated by Isothermal Titration Calorimetry. Langmuir 2013, 29, 857–860. 10.1021/la304532k. 23270307

[ref36] Sugiura Y.; Ikedai K.; Nakano M. High Membrane Curvature Enhances Binding, Conformational Changes, and Fibrillation of Amyloidbeta on Lipid Bilayer Surfaces. Langmuir 2015, 31, 11549–11557. 10.1021/acs.langmuir.5b03332. 26474149

[ref37] Papadopoulou P.; van der Pol R.; van Hilten N.; van Os W.; Pattipeiluhu R.; Arias Alpizar G.; Knol R.; Noteborn W.; Moradi M.; Ferraz M.; Aerts J.; Sommerdijk N.; Risselada H.; Sevink G.; Kros A.; et al. Lipase-mediated selective hydrolysis of lipid droplets in phase separated-liposomes. Adv. Mater. 2024, 36, 2310872 10.1002/adma.202310872. 37988682

[ref38] Keller F.; Heuer A. Chain ordering of phospholipids in membranes containing cholesterol: what matters?. Soft Matter 2021, 17, 6098–6108. 10.1039/D1SM00459J. 34100059

[ref39] Pezeshkian W.; Kn̈ig M.; Wassenaar T.; Marrink S. Backmapping triangulated surfaces to coarse-grained membrane models. Nat. Commun. 2020, 11, 2296 10.1038/s41467-020-16094-y. 32385270 PMC7210967

[ref40] Cooke I.; Kremer K.; Deserno M. Tunable generic model for fluid bilayer membranes. Phys. Rev. E 2005, 72, 011506 10.1103/PhysRevE.72.011506. 16089969

[ref41] Müller M.; Katsov K.; Schick M. Coarse-grained models and collective phenomena in membranes: Computer simulation of membrane fusion. Polymer Physics 2003, 41, 1441–1450. 10.1002/polb.10456.

[ref42] Argudo D.; Bethel N.; Marcoline F.; Grabe M. Continuum descriptions of membranes and their interaction with proteins: Towards chemically accurate models. Biochimica et Biophysica Acta (BBA) - Biomembranes 2016, 1858, 1619–1634. 10.1016/j.bbamem.2016.02.003. 26853937 PMC4877259

[ref43] Daoulas K.; Müller M. Single chain in mean field simulations: quasi-instantaneous field approximation and quantitative comparison with Monte Carlo simulations. J. Chem. Phys. 2006, 125, 184904 10.1063/1.2364506. 17115792

[ref44] Milano G.; Kawakatsu T. Hybrid particle-field molecular dynamics simulations for dense polymer systems. J. Chem. Phys. 2009, 130, 214106 10.1063/1.3142103. 19508055

[ref45] Wang X.; Deserno M. Determining the Pivotal Plane of Fluid Lipid Membranes in Simulations. J. Chem. Phys. 2015, 143, 164109 10.1063/1.4933074. 26520500

[ref46] Baoukina S.; Tieleman D. Computer simulations of lung surfactant. BBA - Biomembranes 2016, 1858, 2431–2440. 10.1016/j.bbamem.2016.02.030. 26922885

