# Supplementary material for: Analyzing Lipid Membrane Defects via a Coarse-Grained to Triangulated Surface Map: The Role of Lipid Order and Local Curvature in Molecular Binding
Source: J Chem Theory Comput. 2024 Mar 27;20(7):2888–900. doi: 10.1021/acs.jctc.4c00082 (PMC11008102; doi:10.1021/acs.jctc.4c00082)
Supplement: Supplementary file 1 — ct4c00082_si_001.pdf [file ct4c00082_si_001.pdf]

Supporting information for: Supporting Information for Analysing Lipid Membrane Defects Via a Coarse-Grained To Triangulated Surface Map: the Role of Lipid Order and Local Curvature in Molecular Binding

Rianne W.I.Van Der Pol,† Bregje W. Brinkmann,‡ and G.J. Agur Sevink*,†

†*Leiden Institute of Chemistry, Leiden University, P.O. Box 9502, 2300 RA Leiden, The Netherlands*

‡*Institute of Environmental Sciences, Leiden University, P.O. Box 9502, 2300 RA Leiden, The Netherlands*

E-mail: a.sevink@chem.leidenuniv.nl

Implementation of the CG2TS projection

To obtain the defects size distributions for various lipid structures, each frame in the trajectory is analyzed separately and subjected to the following 5 steps: (1) Definition of the leaflets. Their number depends on the type of structure used. (2) Converting the molecules that generate a leaflet into a set of points. Each lipid is represented by a single point. For Martini 3, we use the position of the GL2 bead. For each point, a director is subsequently identified, based on the local environment. (3) Next, for each point set, a surface is (re)constructed using the Poisson method. This surface is consequently smoothed to a preset resolution. (4) For each surface, a defect matrix is defined by projecting lipids beads onto the surface, and triangles associated with defects are defined. (5) Defects are clustered and the area per defect is given as output. Next, we provide technical details and settings. The outcome of each step in the protocol is analysed via results for a pure DPPC membrane containing 2858 lipids.

1 Lipid pools corresponding to individual leaflets are obtained using the LeafletFinder implemented in MDAnalysis (`mda`). Care should be taken in dealing with lipids that are positioned away from the average position in a leaflet. A cutoff value of 13 Å is used for lipids that are known not to flip-flop between leaflets on the time scale of our simulation, such as PC lipids. Another cutoff value is considered when lipids with enhanced flip-flop rates are present in a membrane, such as ceramide or diacylglycerol. In that case, leaflets are first determined based on all non-flipping lipids, and flip-flopping lipids within a cutoff of 12Å of a leaflet are added to that leaflet. For Martini 2 and 3, the GL2 or an equivalent bead type is used to convert lipids into a set of point coordinates for individual leaflets.

```
L = mda.LeafletFinder(u, 'name GL2' cutoff=13, pbc=True)
L1 = L.group(0)
```

```
distance = distance_array(flipflop.positions, L1.positions)
flipflop_selection_add_to_L1 = np.where(np.min(distance, axis=1))
```

2 For the Poisson surface reconstruction, we additionally need point orientations. After storing point coordinates as a Open3D (`o3d`) PointCloud for each leaflet, orientations are obtained using a build-in nearest neighbor search routine ($N = 30$). For each point, a plane is fitted through the point and its nearest neighbors, and the vector is determined as the normal to this plane. To ensure that all vectors are consistently oriented in each leaflet, *i.e.* towards the tail of the lipid that the point represents, we perform a rather cumbersome evaluation of their orientation. If the orientation is wrong, the vector is aligned by reversing its direction. Figure S1 shows slices of 3D point-vector clouds before and after the alignment procedure for two representative membrane geometries. It illustrates that the procedure does a proper job, with the exception of a few isolated point-vectors. While this finding suggests that further processing is required, carefully analysis shows that the Poisson method is quite tolerant to such isolated mis-orientations. We thus leave such corrections to future fine tuning of our method. For flat bilayers, a straightforward and efficient method is to check their orientation with respect to the tangent 001 plane.

```
PCD_with_normals = o3d.estimate_normals(PCD, NN=30)
```

3 A Poisson method is applied to determine the continuous scalar indicator function, one inside and zero outside, whose Laplacian equals the divergence of the known vector field at the particle positions.[S1] The initial triangulated mesh is obtained as an isosurface of this reconstructed indicator function. We use the octree search function as implemented in Open3D with a depth of 7 to generate this mesh.

Since the tiles of this initial mesh are generally not evenly sized, we employ an iterative

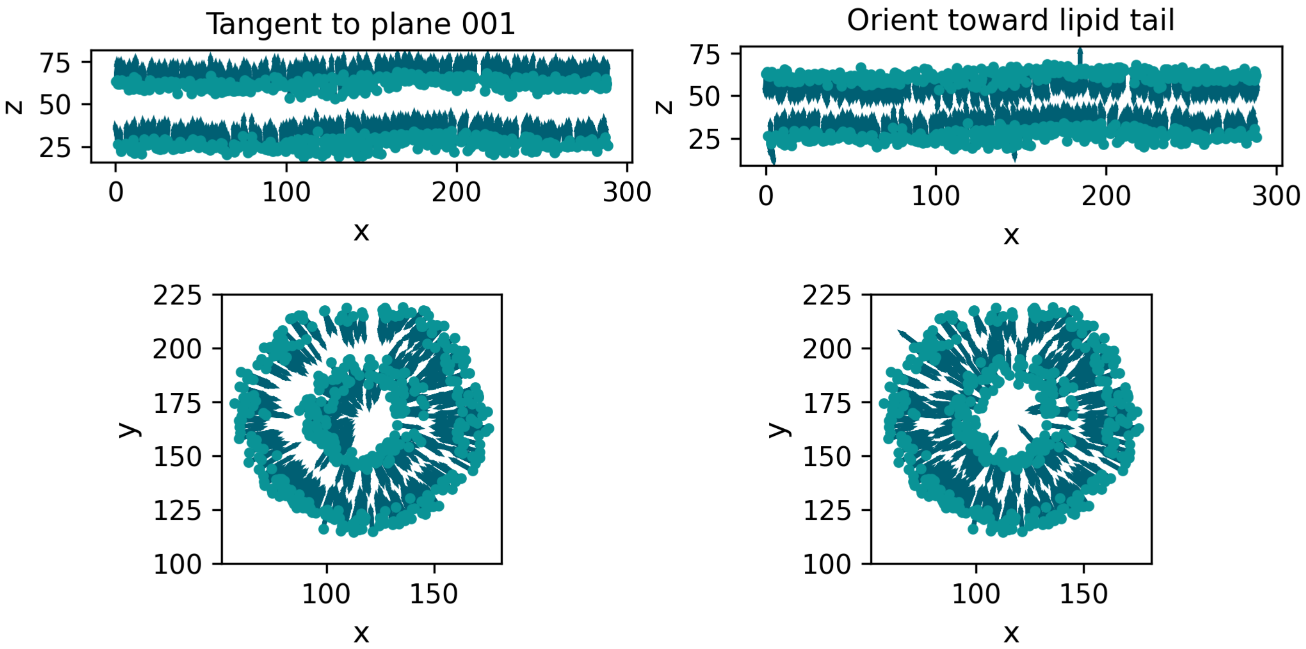

Figure S1: Illustrative 2D slices of the points and vectors output in each of the leaflets (dark and light blue) of steps 1 & 2 for a representative flat and cylindrical membrane, before and after reorientation. (Top) For the flat membrane, normal vectors are efficiently reoriented by minimizing the angle with the (0,0,±1) direction. (Bottom) For cylinders and more complex structures, normal vectors are reoriented towards lipid tails.

Centroidal Voronoi Tesselation (CVT) method to resize and smooth the mesh in two consecutive steps. We employ the protocol of Du et. al[S2] as implemented in the library Optimesh, and note that a two CVT steps procedure is most suited for improving the equilibration performance. The quality of the mesh particularly depends on the number of iterations during the first smoothing step. Figure S2 shows the three meshes obtained after each step for an example case, a DPPC membrane simulated with Martini 3.0, with 2858 lipids.

```
Poisson_Mesh = o3d.create_from_point_cloud_poisson(PCD_with_normals, depth=7)

vertices=Poisson_Mesh.vertices
triangles=Poisson_Mesh.triangles
points, cells = optimesh.optimize_points_cells(vertices, triangles, method='cvt
full', tol=1e-7, number_of_iterations=6)
First_Smoothed_Mesh = o3d.TriangleMesh(points, cells)

First_Smoothed_Mesh_new = o3d.geometry.TriangleMesh.subdivide_midpoint(
   First_Smoothed_Mesh, N_times)
vertices=First_Smoothed_Mesh_new.vertices
triangles=First_Smoothed_Mesh_new.triangles
points, cells = optimesh.optimize_points_cells(vertices, triangles, method="cvt
full", tol=1e-7, number_of_iterations=5)
Second_Smoothed_Mesh = o3d.TriangleMesh(points, cells)
```

For the selected membrane, the distribution of tile sizes for the mesh obtained by the Poisson procedure (in short: Poisson mesh) as well as for the smoothed meshes after each of the two CVT steps are shown in Figure S3. They correspond to the meshes of Figure

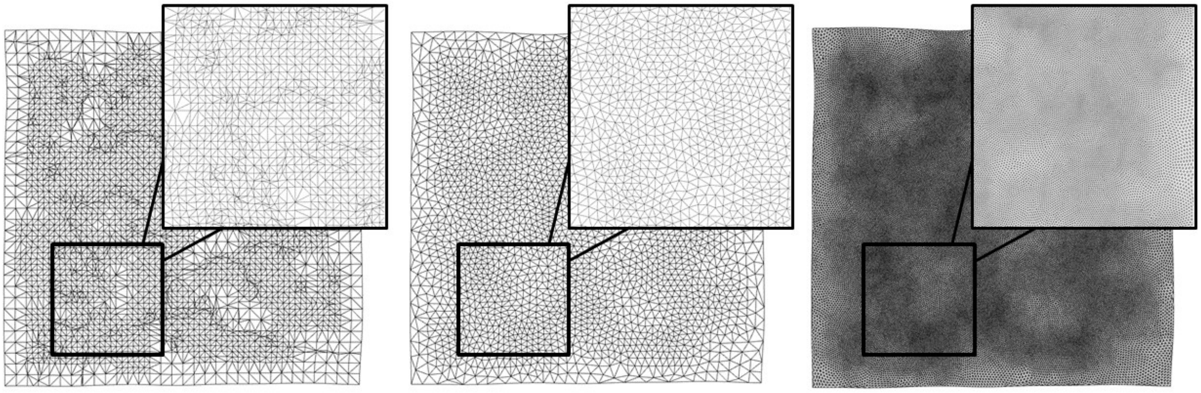

Figure S2: The 2D meshes obtained after surface construction and two smoothing steps: a) the mesh right after surface construction, b) the mesh after the first smoothing and c) the mesh after the second smoothing. Insets are zooms that show mesh detail.

S2. The size distribution for the Poisson mesh, combining a broad spectrum in the lower size range with distinct peaks in the higher range, is clearly not suited for our purpose. It should be noted, however, that these peaks primarily correspond to larger tiles at the edge of the mesh that are generally disregarded in the defect analysis. After the first smoothing step, the size distribution is significantly enhanced, but overall still too broad and with a mean value that is far from the considered target value of 1.0 Å. Next, individual triangles are subdivided (into four smaller ones) until the target area is reached. After smoothing, we obtain a rather narrow size distribution centered around the target value.

The CVT performance is further analysed in Figure S4, which provides details of the iterative improvement of size distributions during the first and the second CVT steps. The first CVT step has the effect of gently narrowing down the size distribution. The resulting distribution, however, is too broad and the average tile size too large, around 60 Å^2, for our purpose. The effect of iterative smoothing in the second CVT step, which includes pre-processing via triangle subdivision, is much less pronounced but still required for obtaining a suitable mesh with an average target tile size of 1.0 Å^2, the default value used in this study. Figure S5 illustrates the ability of the three step procedure to generate a mesh with the target tile size, illustrating that, for all considered target values, the average tile size generated by

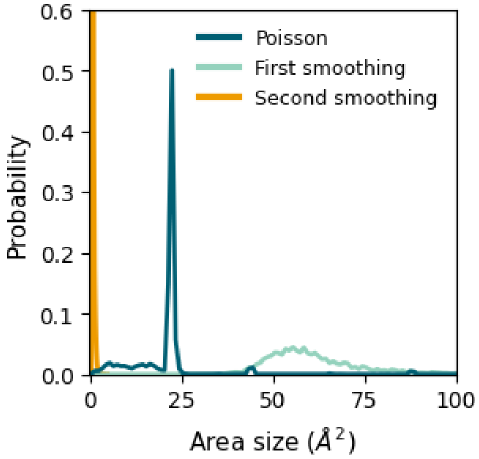

Figure S3: Tile size distribution obtained after the three distinct stages of triangular mesh generation. 1) Initial stage following the Poisson surface reconstruction. At the lower end of the spectrum, tile sizes peak around 25 Å^2, while small peaks at the higher end relate to exceptionally large triangles at the boundary. 2) After the first CVT step, the large spread of the initial mesh has been narrowed down, but the distribution remains rather broad, with an average size that is substantially larger than the target value. 3) After the second step, the distribution has significantly narrowed, and the mean area has reached the target value of 1.0Å

our procedure is slightly smaller than the target value. This is a direct consequence of our rectangular membrane and the fact that triangles can only be subdivided into four smaller ones.

Discretization often introduces a challenge of representability, and our approach is not an exception. In particular, lipid defects of an arbitrary shape have to be represented on a discrete mesh, meaning that both the shape and size of the mesh tiles affect the extracted values. While this representation improves with decreased tile sizes, there is an opposite trend for the associated computational costs. The resulting balance between accuracy and efficiency gives rise to an optimal size unit, which, for the cubic mesh of PackMem, was earlier defined as 1 $\AA$ without much further detail.[S3] Considering our example membrane and calculating defect constants π for different (average) tile sizes in our triangular mesh, see Figure S5, we observe a plateau around 1-1.2 Å^2. Moreover, we should take into account that small tiles improve the description of defect boundaries, and thus lead to (slightly) increased

value for the defect areas or π. Larger tile sizes, on the other hand, introduce an issue for properly capturing connectivity between neighboring defects, and thus increasing the average tile size generally results in smaller defect areas on average or smaller π. Considering this, a target tile size of 1.0 Å^2 was chosen for this study. This has an additional advantage of easing the comparison between TriMesh as CubicMesh results.

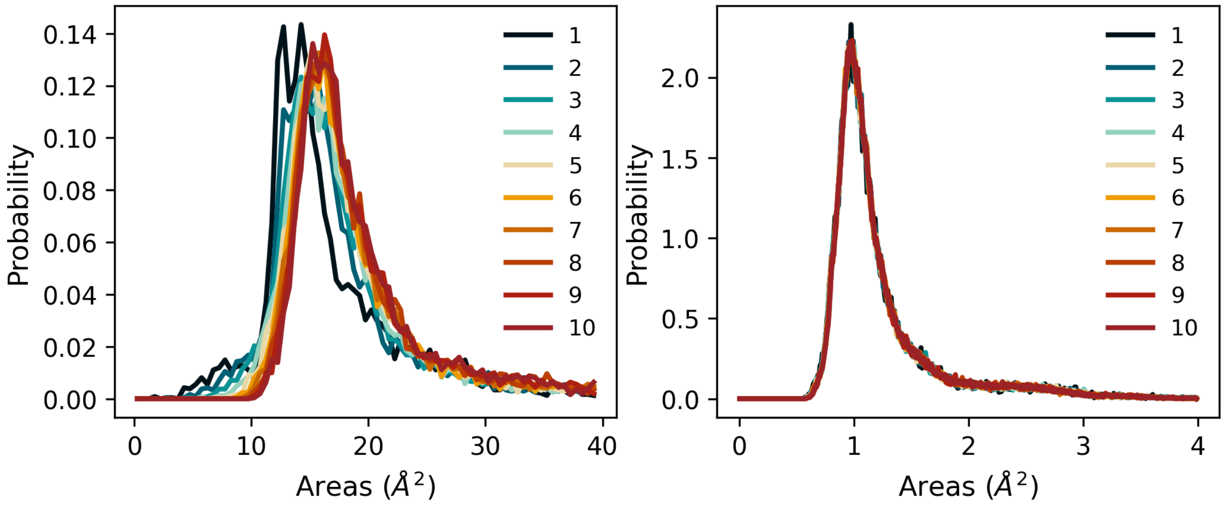

Figure S4: The result of iterative smoothing on the tile size distribution in each CVT step. a) Distribution of tile sizes after each iteration during the first step. Based on this analysis, we performed only 6 iterations for all simulations discussed in the results section. b) Distribution of tile sizes after each iteration during the second step. Only 5 iterations were performed for actual simulations.

Assigning defects

1 For the determination of defect areas within each leaflet, we loop through the set of point coordinates corresponding to that leaflet one-by-one and we identify the lipid corresponding to a selected point coordinate. We project each bead of that lipid onto the surface along the local normal, and we employ a distance constraint to assign tile values. In particular, tiles within a bead-tile distance $(d_{vdw} + d_{triangle})/2$ (center to center) are assigned a fixed value, where d_{vdw} is the van der Waals radius of the projected bead and $d_{triangle}$ the average distance between triangular tile centres. The assigned tile value relates to the chemical nature of the

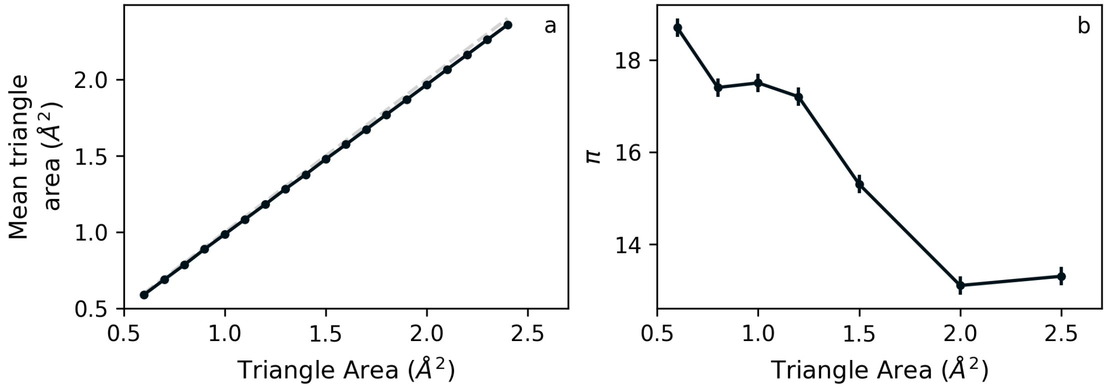

Figure S5: (left) Average tile area obtained by our procedure versus target value. The grey dotted line refers to perfect reproduction of target values. (right) Fitted defect constant π versus the average tile area used in the determination of the defect area distribution. Small tile sizes are preferred to describe irregular defect boundaries, larger tile sizes are computationally efficient but may lead to issues with connectivity. The plateau value around 1 $\AA^2$ is consistent with the tile size of PackMem.

bead. If the bead is aliphatic, the tile value is incremented by 0.001, while for non-aliphatic beads, the increment is 1.0. Once all beads have been considered, tiles with a value of 1 or more are characterised as non-defects. Tiles carrying a value below 1 belong to a defect. All tiles to which no value has been assigned are also considered non-defects, since they are not related to the exposure of hydrophobic membrane domains.

```
d_x = np.sum((grid_coords - bead_position)^2, axis=1)
index_within_radius = np.unique(np.where(d_x <= atom_radius)[0])
M_defects = np.where(np.isnan(M_defects[index_within_radius]), defect_value,
    M_defects[index_within_radius] + defect_value)
```

2 Tiles assigned to defects are used to grow a defect cluster. The cluster grows when the cluster and a new tile share a vertex. For this clustering, we use an implemented method and stitch together all clusters that with a shared vertex. Defects that occupy the boundary of the considered membrane patch, which is subject to periodic boundary conditions, are not considered in our defect analysis for reasons of simplicity. The output of this step is a

normalised distribution of defect areas, $p(A)$, as obtained from computed cluster areas versus their frequency. Since cluster growth depends on the connectivity on the mesh, see Figure S6 for the difference between a square and triangular lattice, the distribution of cluster sizes may be sensitive to the underlying mesh. We expect that this may particularly become an issue close to the critical value for the defect density. Using results from percolation theory, the critical concentration p_c for a percolating cluster is determined by the choice of the (structured) site lattice, varying from $p_c = 0.59275$ for a cubic lattice with $Z = 4$ neighbors to $p_c = 0.5$ for a triangular lattice with $Z = 6$, see Figure S6. The total defect density in our membranes is typically only a few percent, however, so well below the critical value for all considered membranes. The results section indeed shows that the results of our protocol and that of PackMem are consistent.

```
mesh_all_defects = o3d.geometry.TriangleMesh()
mesh_all_defects.triangles = triangles_mesh[M_defects_discrete == defect_value]
labels, clusters, areas = mesh_all_defects.cluster_connected_triangles()
mesh_all_defects.delete_unreferenced_vertices()

for vertex in mesh_all_defects.vertices:
   initial_label= NaN
   for label in labels:
      if (vertex in label.vertices) and (initial_label == NaN):
         initial_label=label.number
      else if (vertex in label.vertices) and (initial_label != NaN):
         merge_labels()
      else:
         continue
```

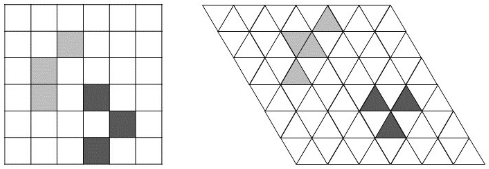

Figure S6: Clustering on a regular square and triangular lattice. In both cases, tiles relating to defects (solid) and non-defects (open) are clustered when they share a vertex. It clearly illustrates the role of the connectivity: each square is surrounded by 8 neighbors, while each triangle is surrounded by 12 neighbors.

Assigning domains based on curvature

As mentioned in the Methods section, one may divide the mesh representing the membrane into domains with negative, zero or positive curvature. In such a case, the exact curvature is not needed, only the sign is sufficient. Since the buckled and the junction membranes of interest feature zero curvature along one of the Cartesian directions (the y-direction) by design, we first project the normal vectors for each tile along this direction, reducing the membrane dimension by one. Yet, projecting the unstructured triangular 2D mesh like this does not generate the desired regular mesh, so we bin and average normal vectors in voxels to generate a structured 2D mesh of vectors. Next, we evaluate the sign of the curvature in each bin by averaging over the two angles between the normal in that bin and the normals of the neighboring bins at either side. Positive and negative curvature relate to values of the averaged angle in the interval $[345^0 \pm 10^0, 15^0 \pm 10^0]$, which may be tweaked depending on the maximum curvature of the mesh, while bins with angles outside this range are automatically assigned to the flat domain. Consequently, we can relate the defect position in the mesh directly to curvature via the binning projection.

Contact fraction

An earlier study[S4] quantified the extent of phase separation, in their case a membrane composed of DPPC, DLiPC and cholesterol or the raft system adopted in this study, via the

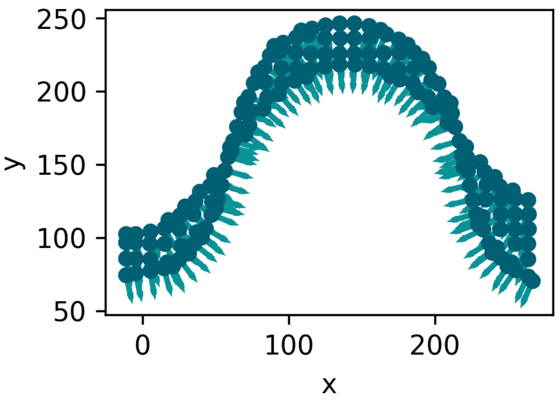

Figure S7: Buckle divided into voxels with each voxel having it's own averaged normal.

contact fraction f_{i-j} between two lipid species i and j, defined as

$$\begin{aligned} f_{DPPC-DLiPC} &= \frac{c_{DPPC-DLiPC}}{c_{DPPC-DLiPC} + c_{DLiPC-DLiPC}} \\ f_{CHOL-DLiPC} &= \frac{c_{CHOL-DLiPC}}{c_{CHOL-DLiPC} + c_{CHOL-DPPC}} \end{aligned} \quad \text{(S1)}$$

Here, c_{i-j} represents the number of contacts between the two lipid species i and j. Although we employ the same distance threshold of 1.1 nm for contacts between lipids,[S4] we introduce an alternative contact fraction. In particular, we redefine f_{i-j} as

$$f_{i-j} = \frac{c_{i-j}}{\rho_j \sum_k c_{i-k}} \quad \text{(S2)}$$

meaning that we normalise by the total number of contacts and the mole fraction ρ_j of lipid specie j. In that way, full demixing gives rise to a vanishing contact fraction, while full mixing provides unity. The reported values in the Result section for the raft system consider DPPC as the reference lipid type.

Curvature

The relation between the defect constant π and global curvature $J = c_1 + c_2$, with $c_i = 1/R_i$ the two principle curvatures and R_i the two principle radii, was previously analysed in the

context of protein binding,[S5,S6] with the most systematic one identifying a linear relation $\pi_J = \pi_0 + \alpha J$ for all five considered lipid compositions in a vesicular or tubular geometry of variable radius (excluding DPPC lipids).[S6] Table S1 shows the value of the two coefficients in the linear fit that was determined from the data extracted from the published $\pi - J$ plots.[S6] To test our protocol, we performed simulations of two additional curved DPPC membrane that are both in the liquid phase, a vesicle ($R_1 = R_2$=13 nm, $J = 0.154$ nm^{-1}) and a tube ($R_1 = 5.5$ nm, $c_2 = 0$, $J = 0.182$ nm^{-1}), see Figure S8. Although this sampling constitutes only a minimal set of three points, they agree with the earlier results in also featuring a distinct linear relation. More quantitative comparison with literature values, however, is often prohibited by particular conditions that have a profound effect on calculated lipid defect constants, for instance temperature and stress. One should be aware that it is not the absolute temperature T that is important - we note that both our CG approach and our $T = 303$ K is comparable to the $T = 300$ K used by Vanni *et al*[S6] - but rather the distance from the critical temperature for phase transition. Reproduction of phase boundaries is a known issue in coarse graining and relates directly to the considered AA-to-CG mapping procedure. Temperature is a factor to be aware of, since the defect constant π was identified to be a distinct function of T, $\pi = \pi(T)$, and a threefold increase in π was observed when going from $T = 300$ to 320 for a DPPC-solvent system, entirely in the fluid phase.[S3] In particular, the gel-to-liquid transition temperature for DPPC was determined as $T_c = 292K$ for Martini CGMD, significantly below the experimental value.[S7] Coefficients quite similar to ours were derived from published data for curved POPC membranes simulated using Martini CGMD. Yet, this study determined π via a 3D-based defect assignment procedure, which was developed, like our approach, to deal with irregularity.[S8] Secondly, it is known that the equilibration of closed lipid membranes poses a general issue due to the very low permeability of lipid membranes to water, making it hard to release pressures due to excess solvent. If not properly dealt with, defect constants may thus feature an additional contribution originating from residual tension in the membrane,[S9] since also tension has been shown to generate

a linear increase of π.[S10] Effective equilibration of such membrane geometries is usually carried out via (artificial) poration, but these pores should be maintained long enough for all membrane stresses to be relieved, which is a subtle issue. The finding that our gradient ($\alpha \approx 226$) is higher than those calculated from reported data of Vanni *et al*[S6] but in the same range as those of Tripathy *et al*,[S8] can thus be explained, see the arguments above, but further investigation of the precise origin, although very interesting, is beyond our current purpose. We note that our results for the buckled fluid membrane show that the π_{J+} is only slightly increased compared to π_{J0} for a flat membrane in that case.

Table S1: Coefficients from a linear fit to the data points extracted from published π-J graphs using the online Plot Digitiser tool.

system	π_0	α	source
POPC	10.2	44.8	Vanni *et al*[S6]
DOPC	11.5	49.9	Vanni *et al*[S6]
DMPC	7.6	22.7	Vanni *et al*[S6]
POPC+DPPC	9.4	43.6	Vanni *et al*[S6]
POPC+POG	11.6	36.5	Vanni *et al*[S6]
POPC	31.7	165.6	Tripathy *et al*[S8]
DPPC	16.9	226.2	this study

The area of exposure

Assuming that molecular binding to a vesicle requires a minimal defect size in the range between the small π^{gel}_{flat} and the larger π^{liquid}_{flat}, we may analyse the role of lipid order in the exposure. We disregard the energetic gain needed for positioning a molecule from the 3D solution to the 2D environment close to a membrane in this analysis. Since $\pi_J > \pi_0$, it follows that a vesicle in the liquid phase is exposed to binding in its entirety, whereas only the curved edges are exposed to binding when the vesicle is in the gel phase. For the experimental study of molecular binding, it thus makes sense to analyse the percentage of the gel vesicle area that is curved.

We assume that the surface of one of the Platonic solids is a proper representation of

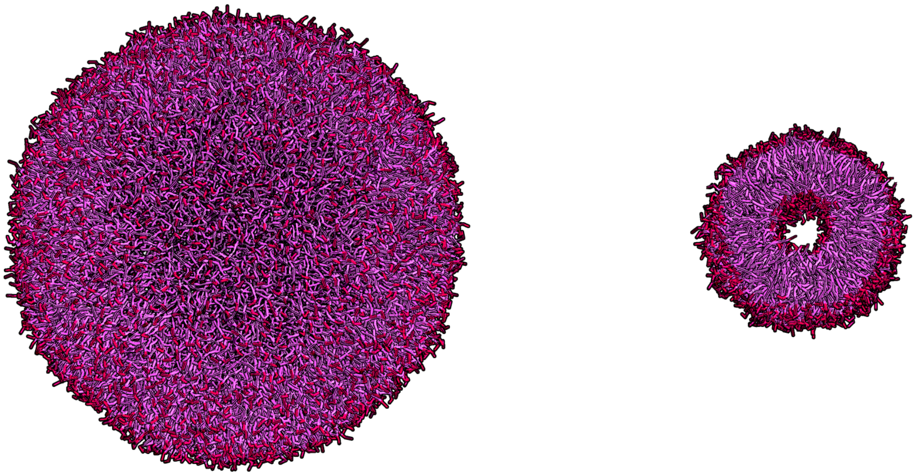

(a) Vesicle and tube

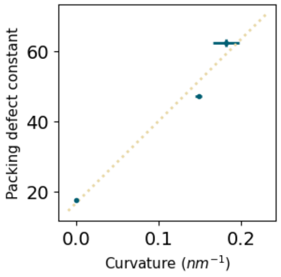

(b) Curvature vs packing defect

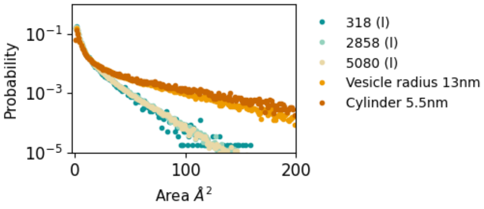

(c) Distribution

Figure S8: The role of curvature. (a) Snapshot of a DPPC vesicle of 13 nm radius, (b) Snapshot of a DPPC tube of 5.5 nm radius, (c) Defect constant π_J (vertical axis) versus curvature $J = c_1 + c_2$ for a flat membrane ($J = 0$ nm^{-1} and $\pi = 17.5 \pm 0.2$), vesicle ($J = 0.148$ nm^{-1} and $\pi = 47.2 \pm 0.5$) and a tube ($J = 0.182$ nm^{-1} and $\pi = 59.4 \pm 1.1$), and (d) Probability distribution (vertical axis) versus area (horizontal axis) for the three structures with different curvature.

the outer membrane of a faceted gel vesicle. The total size of the vesicle determines which Platonic solid we should consider. Since straight angles are not realistic in molecular biostructures, we assume that edges are rounded off to a width of 1 nm. Fixing the total surface area, see table S2 for the relation between surface area and the unit of length a, we may calculate the area of the curved domain, *i.e.* the edge area, for the different shapes, see Table S3. In particular, we consider the surface area of a sphere with a radius R as a reference. We consider $R = 25$ and 100 nm; for the smallest, $R = 25$ nm, the reference surface area is 7854 nm^2. Forming edges is energetically unfavourable, so it is likely that the edge area will be minimal.

Table S2: Geometrical properties of the Platonic solids. These convex, regular polyhedron in three-dimensional Euclidean space provide a reference for faceted 3D vesicles.

Structure	faces	edges	vertices	Surface Area
Tetrahedron	4	6	4	$\sqrt{3}a^2$
Cube	6	12	8	$6a^2$
Octahedron	8	12	6	$2\sqrt{3}a^2$
Dodecahedron	12	30	20	$3\sqrt{5(5+2\sqrt{5})}a^2$
Icosahedron	20	30	12	$5\sqrt{3}a^2$

Table S3: Using a sphere of radius R as a reference, the basis length of the edge and the total edge area are determined and expressed as a percentage of the total surface area.

Structure	R (nm)	Edge a (nm)	Total edge area (nm^2)	%
Tetrahedron	25	67.34	404.03	5.1 %
Cube	25	36.18	434.16	5.5 %
Octahedron	25	47.62	571.39	7.3 %
Dodecahedron	25	19.50	585.13	7.5 %
Icosahedron	25	30.11	903.44	11.5 %
Tetrahedron	100	269.35	1616.13	1.3 %
Cube	100	144.72	1736.64	1.4 %
Octahedron	100	190.46	2285.55	1.8%
Dodecahedron	100	78.02	2340.5	1.9 %
Icosahedron	100	120.46	3613.77	2.9 %

Superposition principle

Suppose that the defect distributions $p_1(A)$ and $p_2(A)$ for the two one-component or one-phase membranes can be fitted by single exponentials $b_1 \cdot \exp(-A/\pi_1)$ and $b_2 \cdot \exp(-A/\pi_2)$ within a certain fitting window $[A_{min}, A_{max}]$. Following the superposition principle, the distribution p_3 for the mixed system is $p_3(A) = \frac{1}{2}(p_1(A) + p_2(A))$, where the prefactor of 1/2 is introduced for normalisation. We assume that $p_3(A)$ can also be fitted well by a single exponential $b_3 \cdot \exp(-A/\pi_3)$ within this window. This provides the relation

$$\ln(\frac{b_1}{2}) - A/\pi_1 + \ln(1 + Be^{-DA}) = \ln(b_3) - A/\pi_3, \tag{S3}$$

where we have taken the logarithm at both sides and defined new variables $B = b_2/b_1$ and $D = 1/\pi_2 - 1/\pi_1 = (\pi_1 - \pi_2)/\pi_2\pi_1$. Extracting the two unknowns, p_3 and π_3, from Eq. S3 enables direct comparison to numerical values for the considered mixed systems in the Results section. Next, we expand $\ln(1 + Be^{-DA})$ into the first two terms of a Taylor series around A_0, a value within the fitting window, to obtain

$$\ln(\frac{b_1}{2} + \frac{b_2}{2}e^{-DA_0}) + \frac{A_0 BD}{e^{DA_0} + B} - A(1/\pi_1 + \frac{BD}{e^{DA_0} + B}) \tag{S4}$$

for the left hand side of Eq. S3. We note that this expression is fully symmetric, meaning that it does not depend on the order of assignment. Finally, we may use the equality of Eq. S3 to compute the defect constant for the mixture as

$$\begin{aligned} \frac{1}{\pi_3} &= \frac{1}{\pi_1} + \frac{BD}{e^{DA_0} + B} \to \pi_3 = \frac{\pi_1(e^{DA_0} + B)}{e^{DA_0} + B(1 + \pi_1 D)} \\ \frac{1}{\pi_3} &= \frac{1}{\pi_2} - \frac{De^{DA_0}}{e^{DA_0} + B} \to \pi_3 = \frac{\pi_2(e^{DA_0} + B)}{B + e^{DA_0}(1 - \pi_2 D)} \end{aligned} \tag{S5}$$

Correlation π to standard membrane descriptors: a literature overview

Since the publication of Packmem, a broad range of membrane compositions has been considered by this method. As it is more common to specify the area per lipid (APL) than the orientational order parameter P_2, we have collected data from various studies to correlate π with common descriptors like the APL. Geissler *et al*[S11] is the only one to provide P_2 as well. Owing to differences in force fields employed in the different studies, which affects the value of π, see discussion in the main text, combining all data for a direct comparison is an issue. Yet, for individual force fields, the limited data suggests that both attributes are likely correlated despite the outliers.

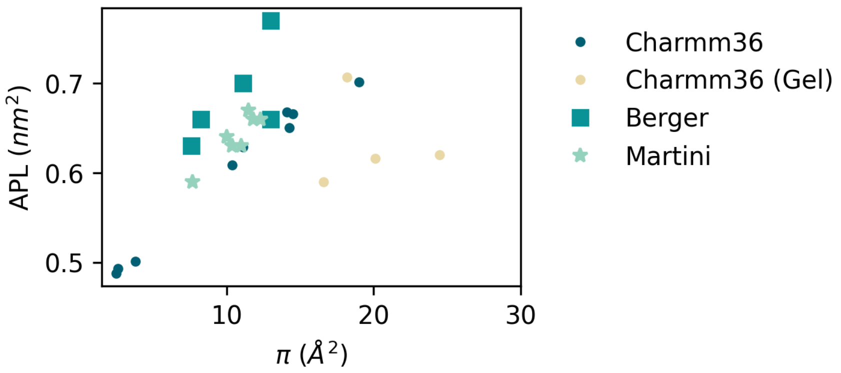

Figure S9: Area per lipid (APL) versus defect constant π. The plotted data was extracted from four studies in the open literature.[S12–S15] Different symbols are used to distinguish between the considered force field (Charmm, Berger and Martini) or phase.

Details of the fitting procedure

In agreement with standard practice, we do not consider the entire range of the defect area distribution in the fitting procedure. Given that the defect area decays exponentially, extensive sampling is required for obtaining a proper signal-to-noise ratio for the largest defect sizes. As a result of natural limitations to sampling, some scatter is usually present in this part of the range, which rationalises the need for a maximum defects size considered in the fit. The smallest defect sizes, on the other hand, primarily reflect the free volume

associated with the packing of hard spheres into a quasi-2D membrane structure. Also the information in this range is thus not relevant for the analysis of defects for particular lipids. This observation is further clarified by a zoom of the smallest 0-6 Å^2 range for a liquid and gel DPPC membrane, see Figure S10. The similarity of this information to the radial distribution function clarifies that this information only relates to order at the smallest scale. Whereas Packmem restricts the lower boundary in the fit to a fixed 15 Å^2, we take a more flexible approach. In particular, we base our fitting range on standard diagnostics for linear regression.

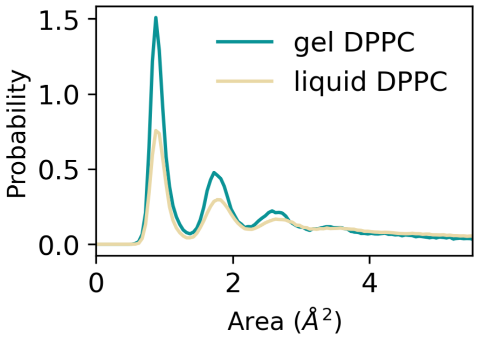

Figure S10: Probability distributions in the lowest defect area range for a gel and liquid DPPC membrane. The peaks, which are higher in the ordered (gel) than in the disordered (liquid) state, clearly show that this information relates to small scale structure rather than defects.

For all flat membranes considered in this study, we used both CM (Packmem) and TM (our protocol) to determine π. A comparison is found in Figure S11. Furthermore, all diagnostics plots upon which the fitting range is determined are given below.

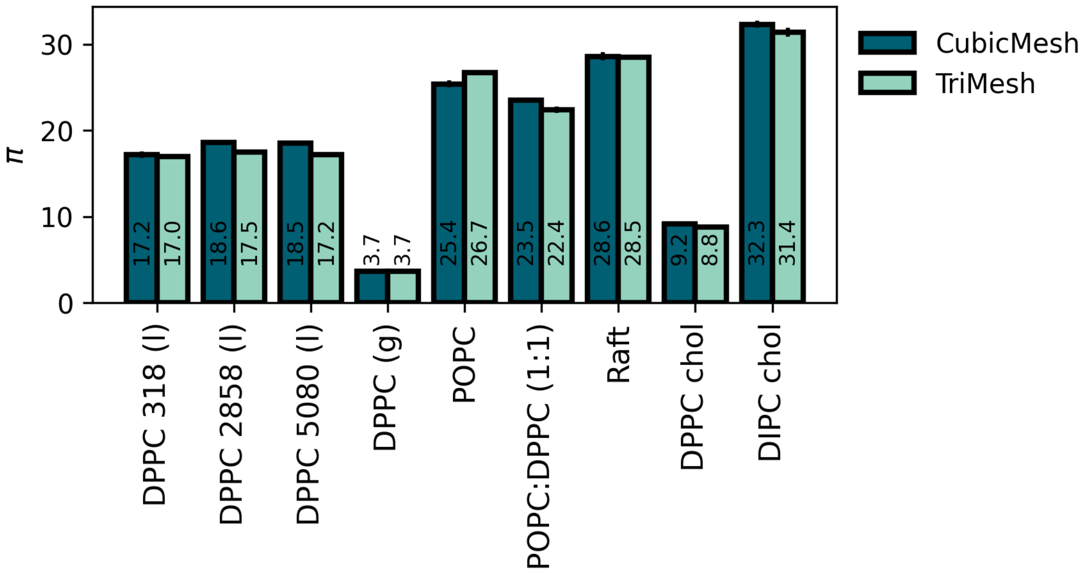

Figure S11: Defect constants π for all flat membranes considered in this study, obtained using the TriMesh (cyan) of this study and the CubicMesh (dark blue) of Packmem.

Table S4: Fitted parameters for all flat membrane simulations, for both the Triangular Mesh as well as the Cubic Mesh. For this comparison, we have considered the best fit for CM instead of the standard range of Packmem.

**Membrane**	CM or TM	π	R^2	min	max	prob min
DPPC 318	CM	17.2 ± 0.4	0.977	25	80	2e-4
DPPC 318	TM	17.0 ± 0.3	0.979	20	100	1e-4
DPPC 2858	CM	18.6 ± 0.2	0.994	30	80	1e-4
DPPC 2858	TM	17.5 ± 0.2	0.995	25	200	2e-4
DPPC 5080	CM	18.5 ± 0.2	0.996	28	100	2e-4
DPPC 5080	TM	17.2 ± 0.1	0.998	28	75	2e-4
DPPC gel	CM	3.7 ± 0.1	0.997	7	20	1e-4
DPPC gel	TM	3.7 ± 0.2	0.992	5.5	23	1e-4
POPC	CM	25.4 ± 0.4	0.990	28	70	3e-4
POPC	TM	26.7 ± 0.3	0.993	30	90	3e-4
DPPC POPC	CM	23.5 ± 0.2	0.994	28	90	1e-4
DPPC POPC	TM	22.4 ± 0.4	0.995	28	100	1e-4
Raft	CM	28.6 ± 0.5	0.985	28	80	4e-4
Raft	TM	28.5 ± 0.3	0.990	25	100	1e-4
DPPC chol	CM	9.2 ± 0.2	0.992	12	40	5e-4
DPPC chol	TM	8.8 ± 0.2	0.991	12	40	5e-4
DLiPC chol	CM	32.3 ± 0.4	0.991	27	100	1e-4
DLiPC chol	TM	31.4 ± 0.5	0.983	27	100	1e-4

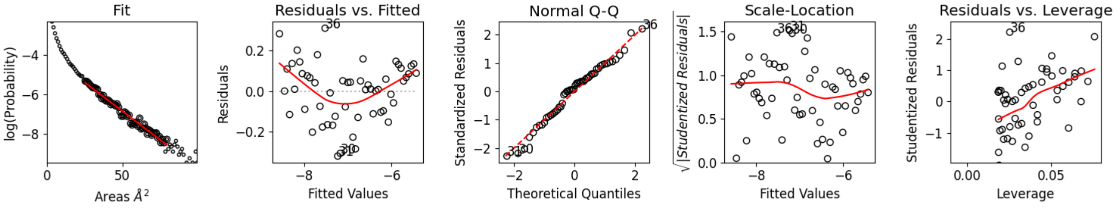

(a) CM

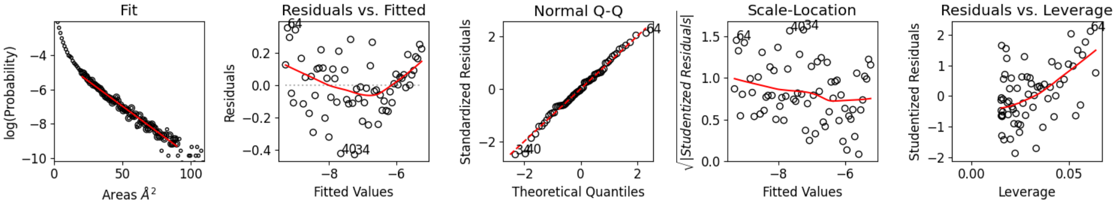

(b) TM

Figure S12: Diagnostic plots for a flat membrane composed of 318 DPPC lipids.

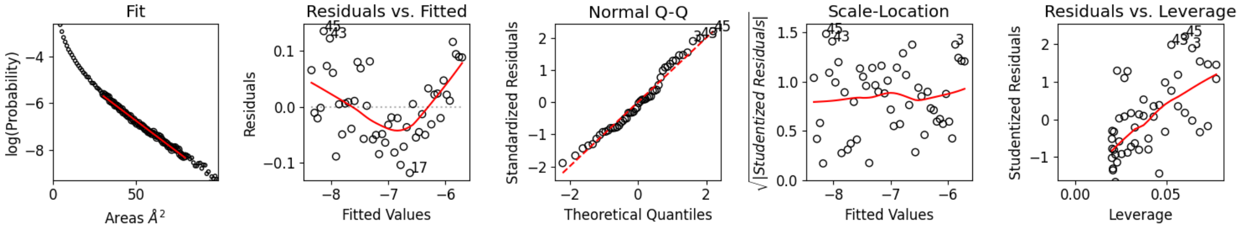

(a) CM

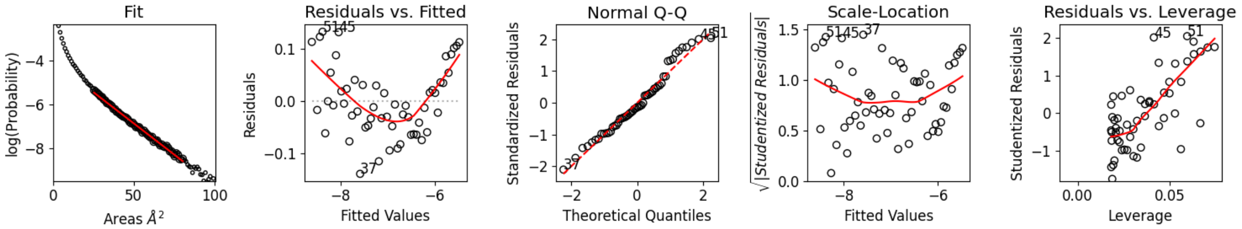

(b) TM

Figure S13: Diagnostic plots for a flat membrane composed of 2858 DPPC lipids.

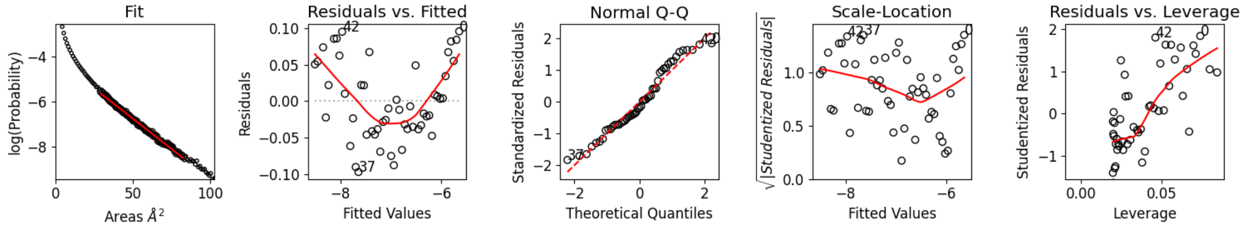

(a) CM

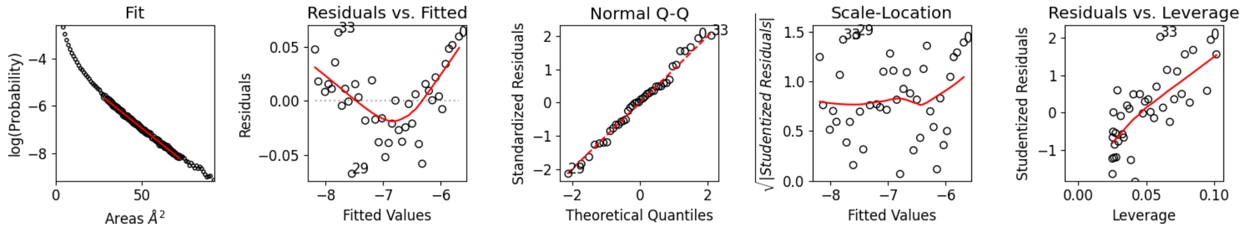

(b) TM

Figure S14: Diagnostic plots for a flat liquid membrane composed of 5080 DPPC lipids.

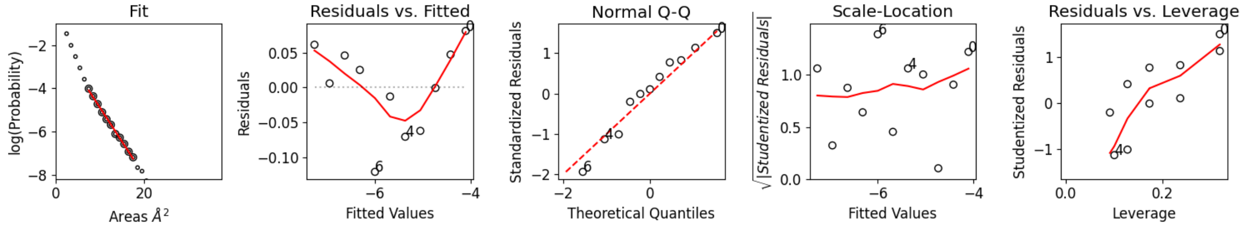

(a) CM

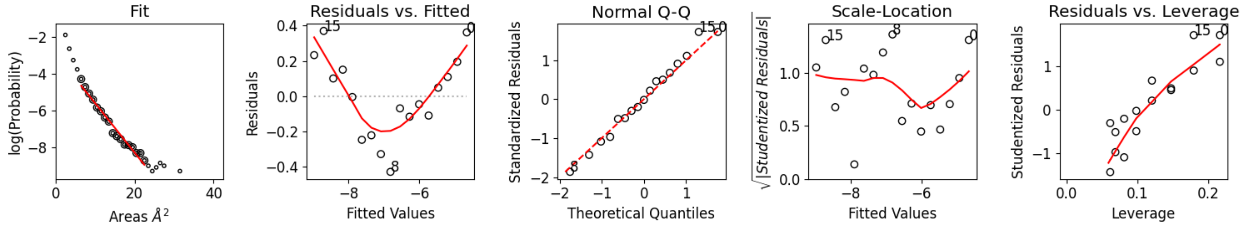

(b) TM

Figure S15: Diagnostic plots for a flat gel membrane composed of 3286 DPPC lipids.

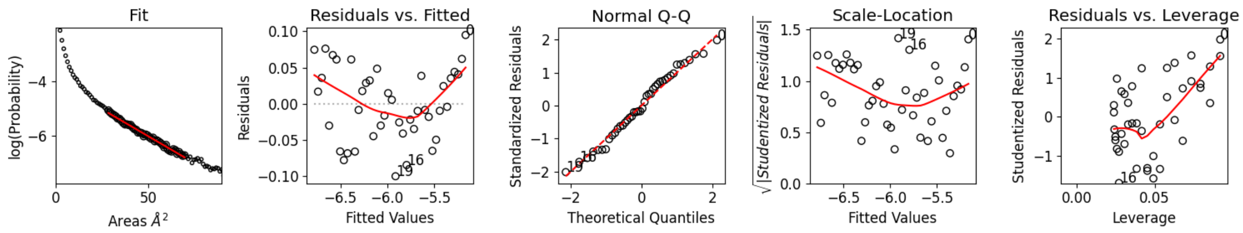

(a) CM

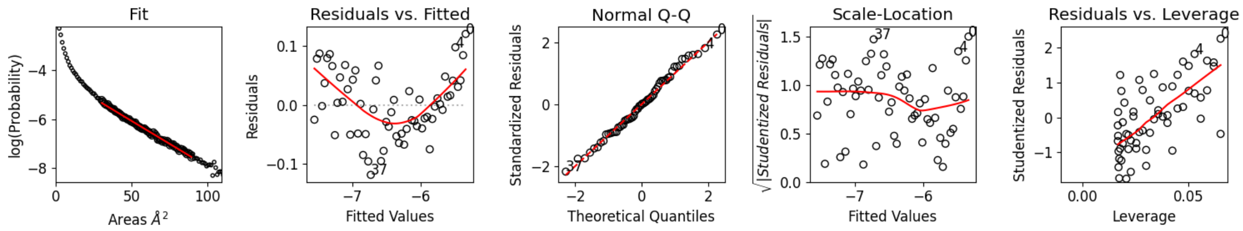

(b) TM

Figure S16: Diagnostic plots for a flat membrane composed of 2858 POPC lipids.

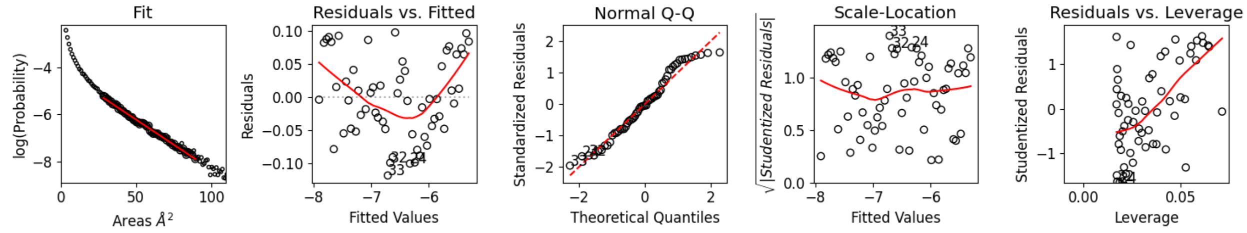

(a) CM

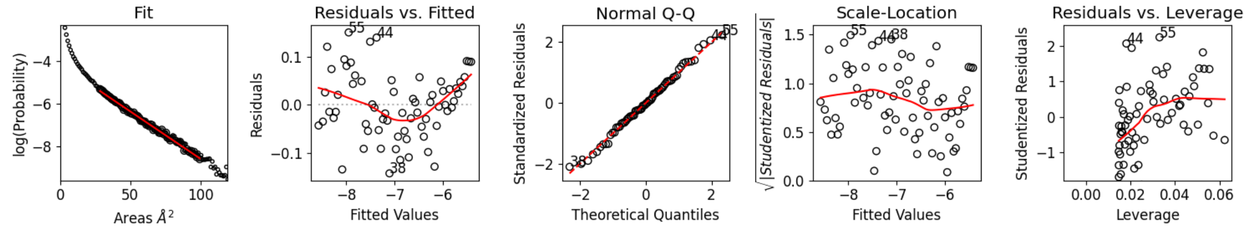

(b) TM

Figure S17: Diagnostic plots for a flat membrane composed of 610 DPPC and 610 POPC lipids (1:1).

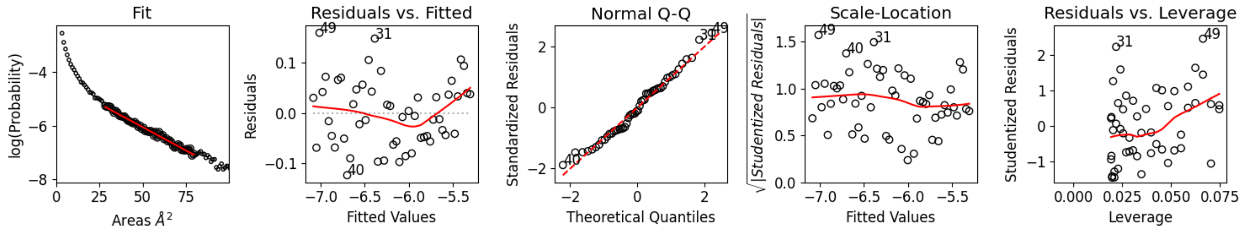

(a) CM

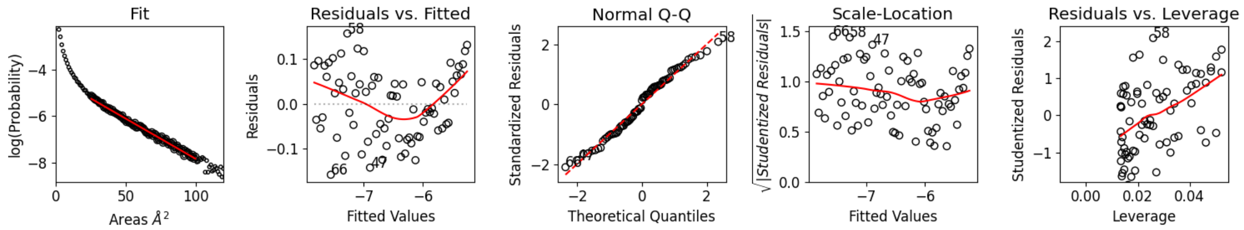

(b) TM

Figure S18: Diagnostic plots for a flat membrane composed of 364 DPPC, 546 DLiPC and 390 cholesterol (raft membrane).

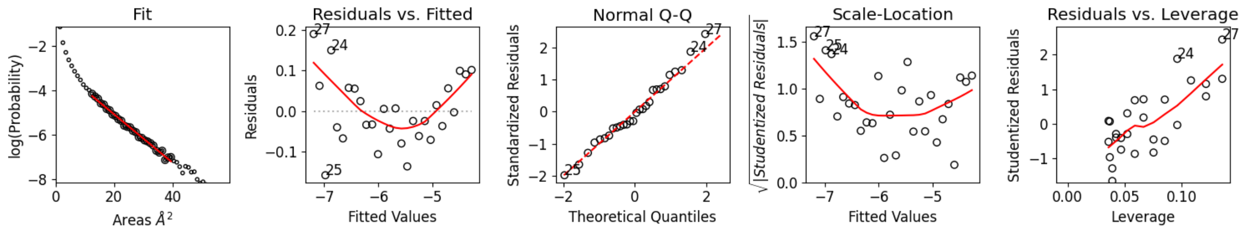

(a) CM

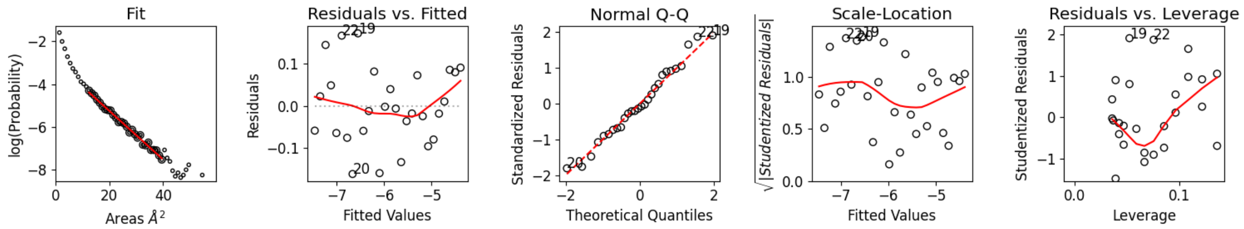

(b) TM

Figure S19: Diagnostic plots for a flat membrane composed of 1164 DPPC lipids and 836 cholesterol.

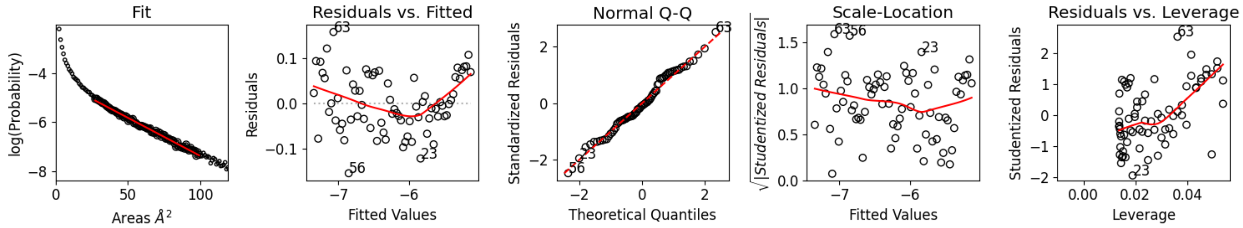

(a) CM

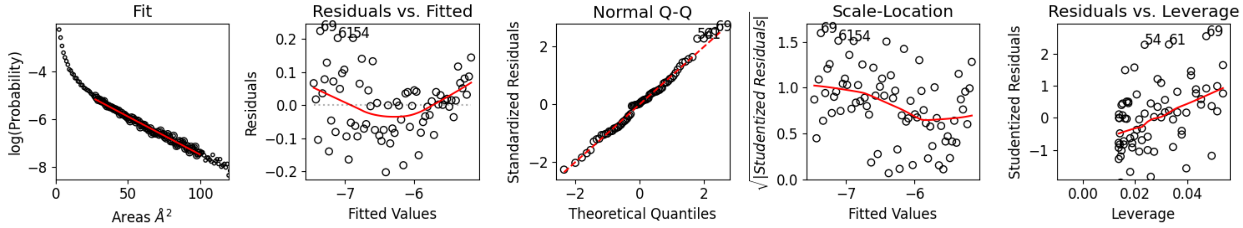

(b) TM

Figure S20: Diagnostic plots for a flat membrane composed of 1668 DLiPC lipids and 332 cholesterol

Table S5: Fitted parameter with used fitting range for all non-flat lipidic structures. All are triangular Mesh.

Membrane	Region	π	b	R^2	min	max	prob min
Buckle 285K	all	34.2 ± 1.3	0.0016	0.905	21.5	90	1e-4
Buckle 285K	+	49.5 ± 0.9	0.0027	0.958	25	186.5	1e-4
Buckle 285K	-	4.2 ± 0.2	0.119	0.995	6.5	16.5	1e-4
Buckle 285K	f	3.9 ± 0.2	0.116	0.997	6.5	16.0	1e-4
Buckle 303K	all	23.5 ± 0.4	0.012	0.984	25	90	1e-4
Buckle 303K	+	28.4 ± 0.5	0.012	0.978	24.5	100	1e-4
Buckle 303K	-	10.5 ± 0.2	0.041	0.992	10.5	50	1e-4
Buckle 303K	f	16.5 ± 0.3	0.0223	0.990	18	60	1e-4
LN		85.9 ± 1.4	0.0045	0.969	50	210	3e-4
Junction	all	40.1 ± 0.7	0.0094	0.971	30	140	2e-4
Junction	+	64.4 ± 2.5	0.0062	0.8733	40	140	2e-4
Junction	-	18.0 ± 0.5	0.0248	0.971	25	80	2e-4
Junction	f	30.2 ± 0.5	0.0134	0.979	30	120	2e-4

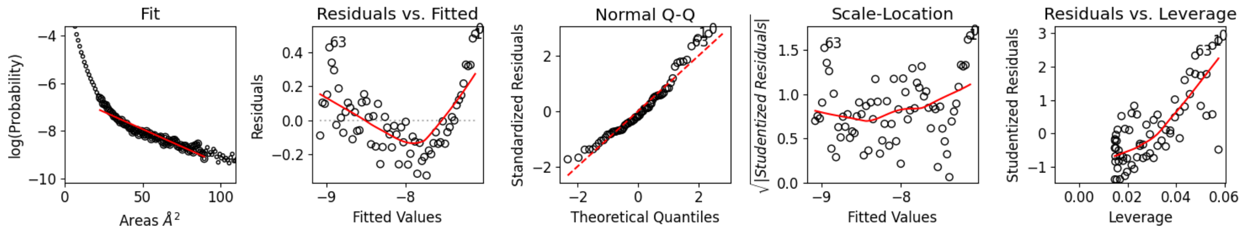

(a) Total

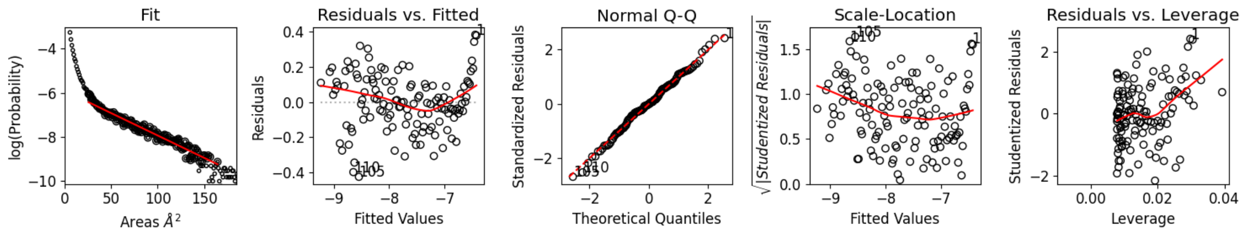

(b) Positive curvature

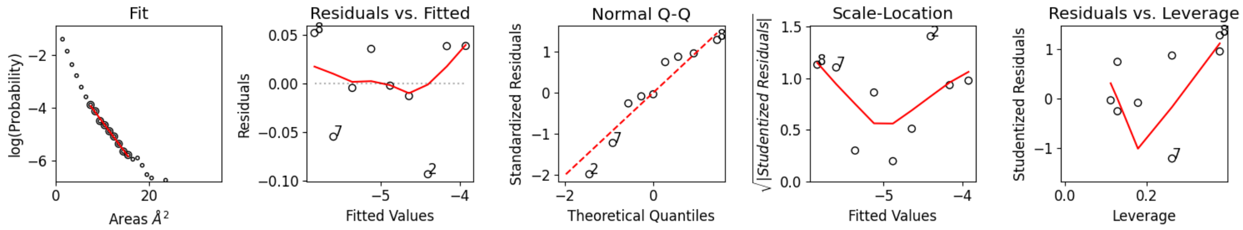

(c) Negative curvature

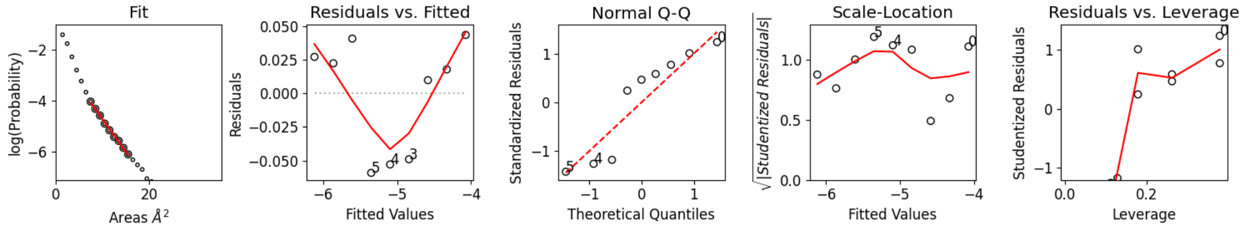

(d) Zero curvature

Figure S21: Buckle 285K (gel)

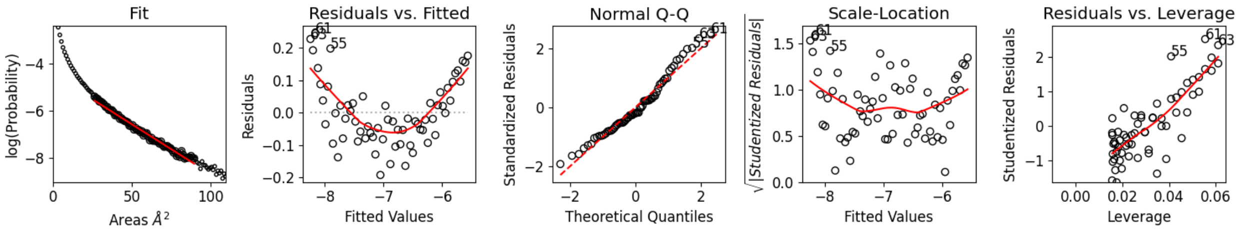

(a) Total

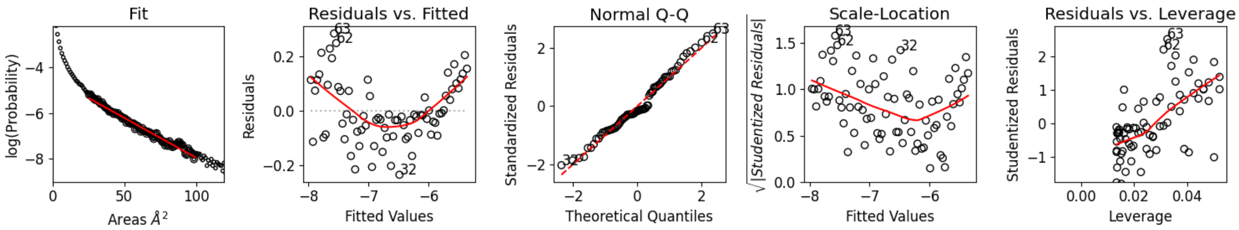

(b) Positive curvature

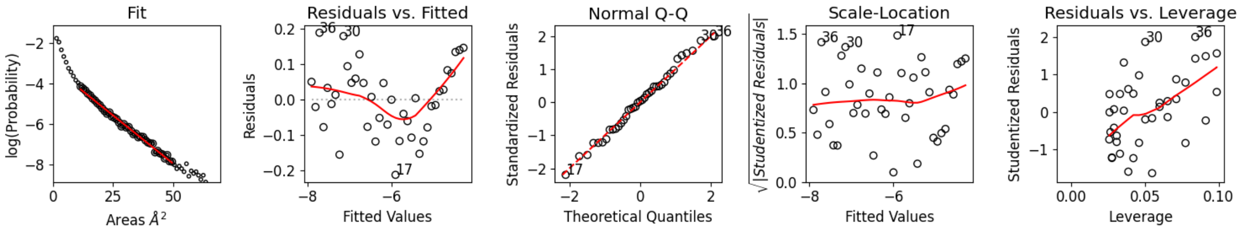

(c) Negative curvature

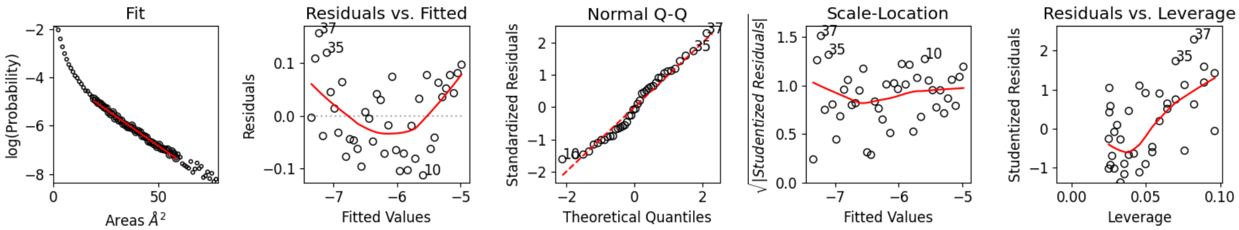

(d) Zero curvature

Figure S22: Buckle 325K (liquid)

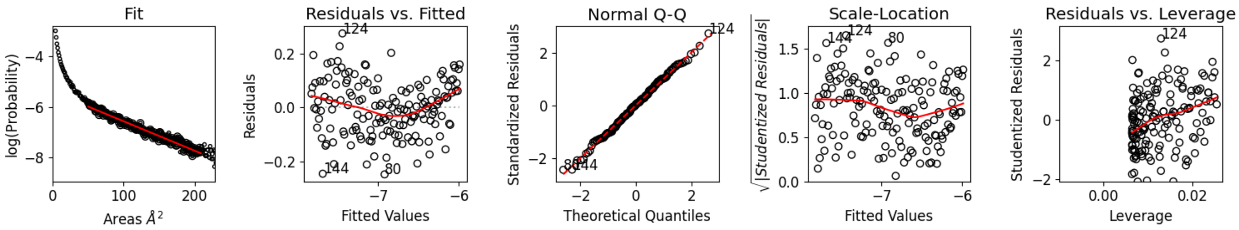

Figure S23: LN

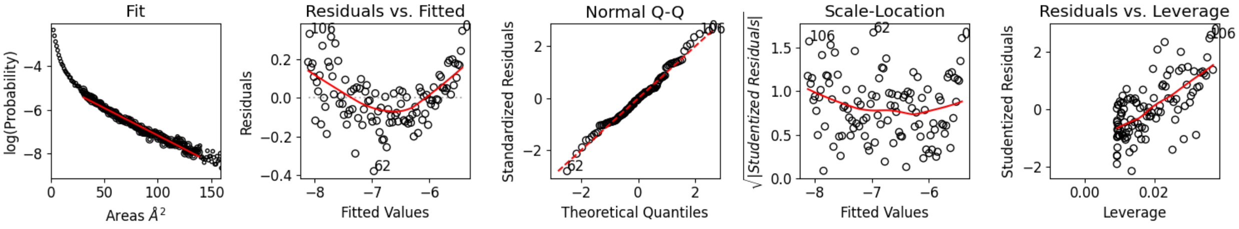

(a) Total

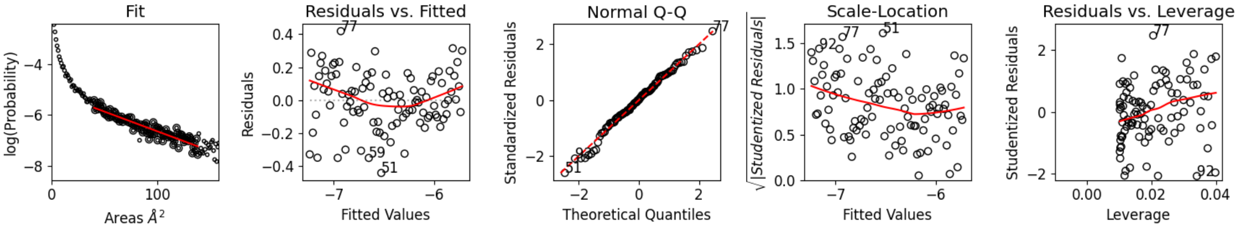

(b) Positive curvature

Figure S24: Junction

References

(S1) Kazhdan, M.; Bolitho, M.; Hoppe, H. Poisson Surface Reconstruction. Symposium on Geometry Processing. 2006.

(S2) Du, Q.; Faber, V.; Gunzburger, M. Centroidal Voronoi Tessellations: Applications and Algorithms. *SIAM Review* **1999**, *41*, 637–676.

(S3) Gautier, R.; Bacle, A.; Tiberti, M.; Fuchs, P.; Vanni, S.; Antonny, B. PackMem: A Versatile Tool to Compute and Visualize Interfacial Packing Defects in Lipid Bilayers. *Biophys. J.* **2018**, *115*, 436–444.

(S4) Liu, Y.; de Vries, A.; Pezeshkian, W.; Marrink, S. Capturing Membrane Phase Separation by Dual Resolution Molecular Dynamics Simulations. *J. Chem. Theory Comput.* **2021**, *17*, 5876–5884.

(S5) Cui, H.; Lyman, E.; Voth, G. Mechanism of membrane curvature sensing by amphipathic helix containing proteins. *Biophys. J.* **2011**, *100*, 1271–1279.

(S6) Vanni, S.; Hirose, H.; Barelli, H.; Antonny, B.; Gautier, R. A sub-nanometre view of how membrane curvature and composition modulate lipid packing and protein recruitment. *Nat. Commun.* **2014**, *5*, 4916.

(S7) Jaschonek, S.; Cascella, M.; Gauss, J.; Diezemann, G.; Milano, G. Intramolecular structural parameters are key modulators of the gel-liquid transition in coarse grained simulations of DPPC and DOPC lipid bilayers. *Biochemical and Biophysical Research Communications* **2018**, *498*, 327–333, Multiscale Modeling.

(S8) Tripathy, M.; Thangamani, S.; Srivastava, A. Three-Dimensional Packing Defects in Lipid Membrane as a Function of Membrane Order. *J. Chem. Theory Comput.* **2020**, *16*, 7800–7816.

(S9) Risselada, H.; Marrink, S. The freezing process of small lipid vesicles at molecular resolution. *Soft Matter* **2009**, *5*, 4531–4541.

(S10) Pinot, M.; Vanni, S.; Ambroggio, E.; Guet, D.; Goud, B.; Manneville, J.-B. Feedback between membrane tension, lipid shape and curvature in the formation of packing defects. *bioRxiv* **2018**, *41*.

(S11) Rogers, J.; Espinoza Garcia, G.; Geissler, P. Membrane hydrophobicity determines the activation free energy of passive lipid transport. *Biophys. J.* **2021**, *120*, 3718–3731.

(S12) Ferru-Clément, R.; Spanova, M.; Dhayal, S.; Morgan, N.; Hélye, R.; Becq, F.; Hirose, H.; Antonny, B.; Vamparys, L.; Fuchs, P. J.; Ferreira, T. Targeting surface voids to counter membrane disorders in lipointoxication-related diseases. *Journal of Cell Science* **2016**, *129*, 2368–2381.

(S13) Vamparys, L.; Gautier, R.; Vanni, S.; Bennett, W.; Tieleman, D.; Antonny, B.; Etchebest, C.; Fuchs, P. Conical Lipids in Flat Bilayers Induce Packing Defects Similar to that Induced by Positive Curvature. *Biophysical Journal* **2013**, *104*, 585–593.

(S14) Vanni, S.; Hirose, H.; Barelli, H.; Antonny, B.; Gautier, R. A sub-nanometre view of how membrane curvature and composition modulate lipid packing and protein recruitment. *Nature Communications* **2014**, *5*.

(S15) Rogers, J.; Espinoza Garcia, G.; Geissler, P. Membrane hydrophobicity determines the activation free energy of passive lipid transport. *Biophysical Journal* **2021**, *120*, 3718–3731.
